# Supplementary material for: Effect of neoadjuvant chemotherapy on tumor immune infiltration in breast cancer patients: Systematic review and meta-analysis
Source: PLoS One. 2023 Apr 27;18(4):e0277714. doi: 10.1371/journal.pone.0277714 (PMC10138237; doi:10.1371/journal.pone.0277714)
Supplement: S1 Appendix — (PDF) [file pone.0277714.s003.pdf]

S1 Appendix
Search strategy:

PubMed and Medline
((humans) AND ((Breast Neoplasm*) OR (Breast Neoplasm))
OR (Neoplasm, Breast)) OR (Breast Tumo*)) OR (Tumour, Breast)) OR (Tumours, Breast)) OR (Tumor, Breast)) OR (Tumors, Breast)) OR (Neoplasms, Breast)) OR (Breast Cancer)) OR (Cancer, Breast)) OR (Mammary Cancer*)) OR (Cancer, Mammary)) OR (Cancers, Mammary)) OR (Malignant Neoplasm of Breast)) OR (Breast Malignant Neoplasm*)) OR (Malignant Tumor of Breast)) OR (Breast Malignant Tumo*)) OR (Cancer of Breast)) OR (Cancer of the Breast)) OR (Mammary Carcinoma, Human)) OR (Carcinoma, Human Mammary)) OR (Carcinomas, Human Mammary)) OR (Human Mammary Carcinomas)) OR (Mammary Carcinomas, Human)) OR (Human Mammary Carcinoma)) OR (Mammary Neoplasms, Human)) OR (Human Mammary Neoplasm*)) OR (Neoplasm, Human Mammary)) OR (Neoplasms, Human Mammary)) OR (Mammary Neoplasm, Human)) OR (Breast Carcinoma*)) OR (Carcinoma, Breast)) OR (Carcinomas, Breast)) OR (Unilateral breast neoplasm*)) OR (Breast Neoplasm, Unilateral)) OR (Unilateral Breast Cancer*)) OR (Breast Cancer, Unilateral)) OR (Breast Neoplasm, Right-Sided)) OR (Right Sided Breast Neoplasm*)) OR (Right-Sided Breast Cancer*)) OR (Breast Cancer, Right-Sided)) OR (Right Sided Breast Cancer*)) OR (Right Sided Breast Neoplasm*)) OR (Right Sided Breast Neoplasm*)) OR (Breast Neoplasm, Left-Sided)) OR (Left Sided Breast Neoplasm*)) OR (Left-Sided Breast Cancer*)) OR (Breast Cancer, Left-Sided)) OR (Left Sided Breast Cancer*))) AND(((((((((((((((((((((((Chemotherapy, adjuvant) OR (Drug therapy, adjuvant)) OR (adjuvant chemotherapy)) OR (adjuvant drug therapy)) OR (Antineoplastic Agents)) OR (Cancer chemotherapy Agent)) OR (Neoadjuvant Therapy)) OR (Neoadjuvant Therapies)) OR (Therapy, Neoadjuvant)) OR (Neoadjuvant Treatment*)) OR (Treatment, Neoadjuvant)) OR (Neoadjuvant Systemic Therap*)) OR (Systemic Therapy, Neoadjuvant)) OR (Therapy, Neoadjuvant Systemic)) OR (Neoadjuvant Systemic Treatment*)) OR (Systemic Treatment, Neoadjuvant)) OR (Treatment, Neoadjuvant Systemic)) OR (Neoadjuvant Chemotherap*)) OR (Chemotherapy, Neoadjuvant)) OR (Neoadjuvant Chemotherapy Treatment*)) OR (Chemotherapy Treatment, Neoadjuvant)) OR (Treatment, Neoadjuvant Chemotherapy)))) AND (((Biomarker*) OR (Biological Marker)) OR (Biologic Marker)) OR (Marker, Biologic*)) OR (Biological Markers)) OR (Biologic Markers)) OR (Markers, Biologic*)) OR (Markers, Immunologic)) OR (Immune Marker*)) OR (Markers, Immune)) OR (Marker, Immun*)) OR (Immunologic Marker*)) OR (Serum Marker*)) OR (Markers, Serum)) OR (Marker, Serum)) OR (Markers, Clinical)) OR (Clinical Marker*)) OR (Marker, Clinical)) OR (Markers, Biochemical)) OR (Marker, Biochemical)) OR (Biochemical Marker*)) OR (Biomarker, Tumo*)) OR (Biomarkers, Tumo*)) OR (Tumour Biomarker*)) OR (Tumor Biomarker*)) OR (Carcinogen Markers)) OR (Markers, Carcinogen)) OR (Markers, Biological Tumo*)) OR (Biological Tumor Marker*)) OR (Biological Tumour Marker*)) OR (Marker, Biological Tumo*)) OR (Biochemical Tumor Marker*)) OR (Biochemical Tumour Marker*)) OR (Tumor Marker, Biochemical)) OR (Tumour Marker, Biochemical)) OR (Tumor Markers, Biologic*)) OR (Tumour Markers, Biologic*)) OR (Biologic Tumor Marker*)) OR (Biologic Tumour Marker*)) OR (Markers, Biologic Tumo*)) OR (Marker, Biologic Tumo*)) OR (Tumor Marker, Biologic)) OR (Tumour Marker, Biologic)) OR (Markers, Biochemical Tumo*)) OR (Marker, Biochemical Tumo*)) OR (Markers, Tumo*)) OR (Biomarkers, Cancer)) OR (Cancer Biomarkers)) OR (Tumor Marker*)) OR (Tumour Marker*)) OR (Marker, Tumo*)) OR (Cancer Biomarker)) OR (Biomarker, Cancer)) OR (Biological Tumor Marker*)) OR (Biological Tumour Marker*)) OR (Membrane Protein*)) OR (Proteins, Membrane)) OR (Surface Protein*)) OR (Proteins, Surface)) OR (Membrane Protein)) OR (Protein, Membrane)) OR (Cell Surface Protein*)) OR (Proteins, Cell Surface)) OR (Protein, Cell Surface)) OR (Surface Protein, Cell)) OR (Cell Membrane Protein*)) OR (Proteins, Cell Membrane)) OR (Membrane Protein, Cell)) OR (Protein, Cell Membrane)))) AND (((Leucocyte*)

OR (Blood Cells, White)) OR (Blood Cell,
White)) OR (White Blood Cell*)) OR (Lymphocyte*)) OR (Lymphoid Cell*)) OR (Cell, Lymphoid)) OR (Cells, Lymphoid)) OR (T-Lymphocyte*)) OR (Thymus-Dependent Lymphocyte*)) OR (Lymphocyte,

Thymus-Dependent)) OR (Thymus-Dependent Lymphocyte*)) OR (T-Cell*)) OR (T Cell*)) OR (Cell, T)) OR (Cells, T)) OR (Lymphocyte, T)) OR (Lymphocytes, T)) OR (B-Lymphocyte*)) OR (B-Cell*)) OR (B Cell*)) OR (B-Lymphocyte)) OR (B Lymphocyte)) OR (Monocyte*)) OR (Killer cell, Natural)) OR (NK Cell*)) OR (Cell, NK)) OR (Cells, NK)) OR (Natural Killer Cell*)) OR (Cell, Natural Killer)) OR (Cells, Natural Killer)) OR (Granulocyte*)) OR (Dendritic Cell*)) OR (Cell, Dendritic)) OR (Cells, Dendritic)) OR (Cell, Interstitial Dendritic)) OR (Cells, Interstitial Dendritic)) OR (Dendritic Cell, Interstitial)) OR (Interstitial Dendritic Cell*)) OR (Dendritic Cells, Interstitial)) OR (Cell, Plasmacytoid Dendritic)) OR (Cells, Plasmacytoid Dendritic)) OR (Cells, Plasmacytoid Dendritic)) OR (Plasmacytoid Dendritic Cell*)) OR (Dendritic Cells, Plasmacytoid)) OR (Basophil*)) OR (Mast Cell*)) OR (Cell, Mast)) OR (Cells, Mast)) OR (Basophils, Tissue)) OR (Basophil, Tissue)) OR (Tissue Basophil*)) OR (Neutrophil*)) OR (Leukocytes, Polymorphonuclear)) OR (Leukocyte, Polymorphonuclear)) OR (Polymorphonuclear Leukocyte*)) OR (Eosinophil*)))) AND ((((((((((((((((((((((((((((((Tumor Microenvironment*) OR (Tumour Microenviroment)) OR (Microenvironment, Tumo*)) OR (Microenvironments, Tumo*)) OR (Cancer Microenvironment*)) OR (Microenvironment, Cancer)) OR (Microenvironments, Cancer)) OR (Lymphocytes, Tumor-Infiltrating)) OR (Lymphocytes, Tumour-Infiltrating)) OR (Tumor Infiltrating Lymphocyte*)) OR (Tumour Infiltrating Lymphocyte*)) OR (Infiltrating Lymphocyte, Tumo*)) OR (Infiltrating Lymphocytes, Tumo*)) OR (Lymphocyte, Tumor Infiltrating)) OR (Lymphocyte, Tumour Infiltrating)) OR (Lymphocytes, Tumor Infiltrating)) OR (Lymphocytes, Tumour Infiltrating)) OR (Tumor- Infiltrating Lymphocyte*)) OR (Tumour-Infiltrating Lymphocyte*)) OR (Lymphocyte, Tumor- Infiltrating)) OR (Lymphocyte, Tumour-Infiltrating)) OR (Tumor-Derived Activated Cell*)) OR (Tumour- Derived Acivates Cell*)) OR (Activated Cell, Tumor-Derived)) OR (Activated Cell, Tumour-Derived)) OR (Activated Cells, Tumor-Derived)) OR (Activated Cells, Tumour-Derived)) OR (Tumor Derived Activated Cell*)) OR (Tumour Derived Activated Cell*)))) AND((Immunohistochemistry) OR (Technique, Immunolabeling)) OR (Techniques, Immunolabeling)) OR (Immunolabeling Techni*)) OR (Technic, Immunolabeling)) OR (Technics, Immunolabeling)) OR (Immunohistocytochemistry)) OR (Immunocytochemistry)) OR (Fluorescent Antibody Technique*)) OR (Antibody Technique, Fluorescent)) OR (Antibody Techniques, Fluorescent)) OR (Technique, Fluorescent Antibody)) OR (Techniques, Fluorescent Antibody)) OR (Immunofluorescence Techni*)) OR (Technic, Immunofluorescence Technics,)) OR (Immunofluorescence Technique,)) OR (Immunofluorescence Techniques,)) OR (Immunofluorescence Fluorescent Antibody Techni*)) OR (Antibody Technic, Fluorescent)) OR (Antibody Technics, Fluorescent)) OR (Technic, Fluorescent Antibody)) OR (Technics, Fluorescent Antibody)) OR (Flow Cytometry)) OR (Flow Cytometr*)) OR (Microfluorometry, Flow)) OR (Flow Microfluorometr*)) OR (Microfluorometries, Flow)) OR (Microfluorimetry, Flow)) OR (Cytofluorometry, Flow)) OR (Cytofluorometries, Flow)) OR (Flow Cytofluorometr*)) OR (Cytometry, Flow)) OR (Cytometries, Flow)) OR (Cell Sortings, Fluorescence-Activated)) OR (Fluorescence-Activated Cell Sorting*)) OR (Sorting, Fluorescence-Activated Cell)) OR (Sortings, Fluorescence-Activated Cell)) OR (Gene expression Profiling)) OR (Transcriptome Analysis)) OR (Gene Expression Profilings)) OR (Profiling, Gene Expression)) OR (Profilings, Gene Expression)) OR (Transcriptome Profiling)) OR (Profiling, Transcriptome)) OR (Profilings, Transcriptome)) OR (Transcriptome Profilings)) OR (Transcript Expression Analysis)) OR (Analyses, Transcript Expression)) OR (Analysis, Transcript Expression)) OR (Transcript Expression Analyses)) OR (Transcriptome Analys*)) OR (Analyses, Transcriptome)) OR (Analysis, Transcriptome)) OR (Gene Expression Monitoring*)) OR (Monitoring, Gene Expression)) OR (Monitorings, Gene Expression)) OR (Gene Expression Pattern Analysis)))))) NOT((Animals OR Review))

EMBASE

Query(breast AND tumor OR (breast AND gland AND tumor) OR (gland AND tumour AND breast AND mass) OR (breast AND neoplasms) OR (breast AND tumour) OR (female AND breast AND neoplasm) OR (female AND breast AND tumor) OR (female AND breast AND tumour) OR (mamma AND tumor) OR (mamma AND tumour) OR (mammary AND gland AND tumor) OR (mammary AND gland AND tumour) OR (mammary AND neoplasms) OR (mammary AND tumor) OR (mammary AND tumor AND cell) OR (mammary AND tumour) OR (mammary AND tumour AND cell) OR (unilateral AND breast AND neoplasms)) AND (leukocyte OR (human AND leucocyte) OR (human AND leukocyte) OR leucocytes OR leukocytes OR (peripheral AND blood AND leucocyte) OR (peripheral AND blood AND leukocyte) OR (peripheral AND leucocyte) OR (peripheral AND leukocyte) OR wbc OR (white AND blood AND cell) OR (white AND blood AND corpuscle) OR (white AND cell) OR (blood AND lymphocyte) OR (f1 AND lymphocyte) OR (immune AND competent AND cell) OR (immune AND lymphocyte) OR (immune AND lymphoid AND cell) OR immunocyte OR (large AND lymphocyte) OR (lymph AND cell) OR (lymphocyte AND f1) OR (lymphocyte AND kinetics) OR lymphocyte OR (immune AND lymphocytes) OR (memory AND lymphocyte) OR (small AND lymphocyte) OR (t AND lymphocyte) OR (amplifier AND t AND lymphocyte) OR (lymphocyte, AND thymus) OR ('suppressor inducer' AND 't lymphocytes') OR (t AND cell) OR (t AND cells) OR 't lymphocytes' OR ('t lymphocytes,' AND 'suppressor inducer') OR (thymic AND lymphocyte) OR (thymus AND dependant AND lymphocyte) OR (thymus AND dependent AND cell) OR (thymus AND dependent AND lymphocyte) OR (thymus AND derived AND cell) OR (thymus AND derived AND lymphocyte) OR (thymus AND lymphocyte) OR (b AND lymphocyte) OR (b AND cell) OR 'b lymphocytes' OR (bone AND marrow AND derived AND lymphocyte) OR (bone AND marrow AND lymphocyte) OR (bursa AND derived AND lymphocyte) OR (lymphocyte, AND b) OR (lymphocyte AND bone AND marrow AND derived) OR (lymphocyte, AND bursa AND derived) OR monocyte OR monocytes OR (monocytes, AND activated AND killer) OR (natural AND killer AND cell) OR (cell AND strain AND nk) OR (cell, AND nk) OR (k AND cell, AND natural) OR (killer AND cell, AND natural) OR (killer AND cells, AND natural) OR (natural AND killer AND activity) OR (natural AND killer AND cell AND activity) OR (natural AND killer AND cells) OR (nk AND cell) OR granulocyte OR (granular AND leucocyte) OR (granular AND leukocyte) OR (granulocyte AND colony) OR granulocytes OR (granulocytic AND leucocyte) OR (granulocytic AND leukocyte) OR (granuloid AND cell) OR (leucocyte AND granule) OR (leucocyte AND polynuclear) OR (leucocyte, AND granulocyte) OR (leukocyte, AND granulocyte) OR (dendritic AND cell) OR (dendritic AND cells) OR (epidermis AND dendritic AND cell) OR basophil OR (basophil AND cell) OR (basophil AND granulocyte) OR (basophil AND leucocyte) OR (basophil AND leukocyte) OR (basophilic AND cell) OR (basophilic AND granulocyte) OR (basophilic AND leucocyte) OR (basophilic AND leukocyte) OR basophils OR (cell, AND basophil) OR (granulocyte,AND basophil) OR (mast AND cell) OR (cell, AND mast) OR labrocyte OR (mast AND cell AND depletion) OR (mast AND cells) OR mastocyte OR neutrophil OR (granulocyte, AND neutrophil) OR (leucocyte, AND neutrophil) OR (leukocyte, AND neutrophil) OR neutrocyte OR neutrocytes OR (neutrophil AND granulocyte) OR (neutrophil AND leucocyte) OR (neutrophil AND leukocyte) OR (neutrophilic AND granulocyte) OR (neutrophilic AND leucocyte) OR (neutrophilic AND leukocyte) OR neutrophils OR (pmn AND granulocyte) OR (pmn AND leucocyte) OR (pmn AND neutrophil) OR (polymorphonuclear AND granulocyte) OR (polymorphonuclear AND leucocyte) OR (polymorphonuclear AND leukocyte) OR (polymorphonuclear AND neutrophil) OR (polymorphous AND leucocyte) OR (polymorphous AND leukocyte) OR (polynuclear AND leucocyte) OR (polynuclear AND leukocyte) OR eosinophil OR (acidophil AND granulocyte) OR (acidophil AND leucocyte) OR (acidophil AND leukocyte) OR

(eosinophil AND granulocyte) OR (eosinophil AND leucocyte) OR (eosinophil AND leukocyte) OR (eosinophile AND granulocyte) OR (eosinophilic AND granulocyte) OR (eosinophilic AND leucocyte) OR (eosinophilic AND leukocyte) OR eosinophils OR (eosinophyl AND leucocyte) OR (eosinophyl AND leukocyte) OR (eosinophylic AND granulocyte) OR (granulocyte, AND eosinophil) OR (leucocyte, AND eosinophil) OR (leukocyte, AND eosinophil)) AND ('chemotherapy, adjuvant'/exp OR 'chemotherapy, adjuvant' OR (chemotherapy, AND ('adjuvant'/exp OR adjuvant)) OR (adjuvant AND chemotherapy) OR (chemotherapy, AND adjuvant) OR (neoadyuvant AND therapy) OR (neoadyuvant AND chemotherapy)) AND (immunohistochemistry OR (antigen AND staining) OR (histochemistry, AND immune) OR immunostaining OR (staining, AND antigen) OR immunofluorescence OR i.f OR (immune AND fluorescence) OR (immune AND fluorescence AND technique) OR (immunofluorescence AND technique) OR (immunofluorescent AND technique) OR (indirect AND immunofluorescence) OR (indirect AND fluorescent AND antibody AND technique) OR (fluorescent AND antibody AND technique, AND indirect) OR (indirect AND fluorescent AND antibody AND test) OR (indirect AND fluorescent AND technique) OR (flow AND cytometry) OR (cytometry, AND flow) OR (flow AND cytofluorometry) OR (gene AND expression AND profiling) OR (gene AND expression AND profile) OR (gene AND product AND profiling) OR transcriptome OR transcriptomes) AND ('tumor microenvironment' OR (cancer AND microenvironment) OR (lymphocytes, AND 'tumor infiltrating') OR (tumor AND infiltrate) OR (tumour AND infiltrate) OR (immune AND infiltrate)) AND [female]/lim AND [humans]/lim

BVS

((tw:(Neoplasias de la Mama)) OR (tw:(Cancer de Seno)) OR (tw:(Carcinoma Mamario Humano)) OR (tw:(Carcinoma de Mama)) OR (tw:(Carcinoma de la Mama)) OR (tw:(Carcinomas Mamarios Humanos)) OR (tw:(Carcinomas de Mama)) OR (tw:(Cáncer Mamario)) OR (tw:(Cáncer de Mama)) OR (tw:(Cáncer de la Mama)) OR (tw:(Cánceres de Mama)) OR (tw:(Neoplasia Maligna de Mama)) OR (tw:(Neoplasia Maligna de la Mama)) OR (tw:(Neoplasia Mamaria Humana)) OR (tw:(Neoplasias Malignas de Mama)) OR (tw:(Neoplasias Mamarias)) OR (tw:(Neoplasias Mamarias Humanas)) OR (tw:(Neoplasía Mamaria)) OR (tw:(Neoplasía de la Mama)) OR (tw:(Tumor Maligno de la Mama)) OR (tw:(Tumor de Mama)) OR (tw:(Tumor de Seno)) OR (tw:(Tumor de la Mama)) OR (tw:(Tumores Malignos de la Mama)) OR (tw:(Tumores Mamarios)) OR (tw:(Tumores de Mama)) OR (tw:(Tumores de Seno)) OR (tw:(Tumores de la Mama))) AND ((tw:(quimioterapia)) OR (tw:(quimiotratamiento))) AND ((tw:(Microambiente Tumoral)) OR (tw:(Linfocitos Infiltrantes de Tumor))) AND ((tw:(Linfocitos B)) OR (tw:(Célula B)) OR (tw:(Células B)) OR (tw:(Células Asesinas Naturales)) OR (tw:(Linfocitos)) OR (tw:(Linfocitos T)) OR (tw:(Célula T)) OR (tw:(Células T)) OR (tw:(Linfocitos T Citotóxicos)) OR (tw:(Linfocitos T CD4-positivos)) OR (tw:(Linfocitos T CD8-positivos)) OR (tw:(Linfocitos T Reguladores)) OR (tw:(Leucocitos)) OR (tw:(Neutrófilos)) OR (tw:(Leucocitos Mononucleares)) OR (tw:(Granulocitos)) OR (tw:(Eosinófilos)) OR (tw:(Basófilos)) OR (tw:(Mastocitos)) OR (tw:(Mastocitos)) OR (tw:(Macrófagos)) OR (tw:(Monocitos)) OR (tw:(Células Dendríticas))) AND ((tw:(Citometría de Flujo)) OR (tw:(Inmunohistoquímica)) OR (tw:(Técnica del Anticuerpo Fluorescente Directa)) OR (tw:(Técnica del Anticuerpo Fluorescente indirecta)) OR (tw:(Transcriptoma)) OR (tw:(RNA-Seq))) AND NOT (Animales) AND NOT (Revisiones)

CENTRAL

((((((((((((((((((Breast Neoplasms) OR Breast Tumors)) OR (Breast Tumours)) OR (Mammary Cancer)) OR (Breast Malignant Neoplasms)) OR (Malignant Tumor of Breast)) OR (Breast Malignant Tumour)) OR (Human Mammary Carcinomas)) OR (Human Mammary Neoplasms)) OR (Breast Carcinoma)) OR (unilateral breast neoplasms)) OR (Unilateral Breast Cancer)) OR (Right-Sided Breast Neoplasm)) OR

(Right-Sided Breast Cancer)) OR (left sided breast neoplasms)) OR (Left-Sided Breast Cancer)) AND ((((((((Chemotherapy, adjuvant) OR (Drug Therapy, adjuvant)) OR (adjuvant chemotherapy)) OR (adjuvant drug therapy)) OR (Antineoplastic Agents)) OR (Cancer Chemotherapy Agent)) OR (neoadjuvant therapy)) OR (neoadjuvant chemotherapy))) AND (((((((((((((((Biomarkers) OR (Biological Markers)) OR (Immunological markers)) OR (Immune markers)) OR (Biomarkers Tumor)) OR (Biological Tumor Markers)) OR (Biomarkers Cancer)) OR (Membrane Proteins)) OR (Surface Protein)) OR (Cell surface proteins)) OR (tumor microenvironment)) OR (cancer microenvironment)) OR (Lymphocytes Tumor Infiltrating)) OR (Tumor infiltrate)) OR (Tumour infiltrate)) OR (Immune infiltrate))) AND (((((((((((Leucocyte) OR (Lymphocyte)) OR (T-Lymphocyte)) OR (B-Lymphocyte)) OR (Monocyte)) OR (Killer Cell, Natural)) OR (Granulocyte)) OR (Dendritic Cell)) OR (Basophil)) OR (Mast Cell)) OR (Neutrophil)) OR (Eosinophil))) AND (((((((Immunohistochemistry) OR (Immunolabeling Technic)) OR (Fluorescent Antibody Technique)) OR (Immunofluorescence)) OR (Flow Cytometry)) OR (Gene expression profiling)) OR (Transcriptome Analysis)) NOT (Review OR Animals)
