# Supplementary material for: Effect of neoadjuvant chemotherapy on tumor immune infiltration in breast cancer patients: Systematic review and meta-analysis
Source: PLoS One. 2023 Apr 27;18(4):e0277714. doi: 10.1371/journal.pone.0277714 (PMC10138237; doi:10.1371/journal.pone.0277714)
Supplement: S2 Appendix — (PDF) [file pone.0277714.s004.pdf]

LEGEND OF FIGURE S1.

Figure S1. **Risk of bias by article**. Review authors' judgments about each risk of bias item for each included study. a) The study question and/or objective of the study is clear. b) Eligibility/selection criteria for the study population are specified and described in advance. c) Study participants were representative of the test/service/intervention in the general or clinical population of interest. d) Eligible participants who met the pre-specified entry criteria were enrolled. e) The sample size was sufficiently large to provide confidence in the findings. f) The test/service/intervention was clearly described and uniformly administered across the study population. g) Outcome measures were pre-specified, clearly defined, valid, reliable, and consistently assessed across all study participants. h) Individuals assessing outcomes were blinded to participants' exposures/interventions i) Loss to follow-up after baseline was 20% or less and loss to follow-up was accounted for in the analysis. j) Statistical methods for changes in outcomes from pre to post intervention were examined. Statistical tests were performed that provided p values for pre- to post-intervention changes. k) Whether an ethics committee approved the protocol, or whether patients signed an informed consent form is clearly described. l) The antibodies, clones and brand used in the labeling were described (For the technique that applies). m) Results were clearly presented.
