# Supplementary material for: Effect of neoadjuvant chemotherapy on tumor immune infiltration in breast cancer patients: Systematic review and meta-analysis
Source: PLoS One. 2023 Apr 27;18(4):e0277714. doi: 10.1371/journal.pone.0277714 (PMC10138237; doi:10.1371/journal.pone.0277714)
Supplement: S1 Table — (PDF) [file pone.0277714.s005.pdf]

Table S1. Articles included and parameters measured	
**Author and year**	**Cells or markers measured**
Abdel-Fatah 2014 [1]	TILs
Abdelrahman, 2021 [2]	PD-L1, FoxP3 and TILs
Alhesa 2022 [3]	Stromal TILs and PD-L1
Chan 2014 [4]	CD8, Foxp3 and CD8/Foxp3 ratio.
Demaria 2001 [5]	Stromal TILs, Intratumoral TILs, CD3 and CD8
Demir 2013 [6]	FoxP3
Dieci 2020 [7]]	PD-L1, CD8, FOXP3 and TILs
García-Martínez 2014 [8]	CD4, CD8, CD3, CD20, FOXP3 and CD68
Graeser 2021 [9]	Stromal TILs, CD4, CD8, PD1 and PD-L1
Grecco-Hoffman 2021 [10]	Stromal TILs and PD-L1
Hee Park 2020 [11]	TILs
Hornychova 2008 [12]	CD3, CD68, CD83, CD56, S100 and CD1a
Kaewkangsadan 2016 [13]	Stromal and intratumoral: TILs, CD4, CD8, FOXP3, CTLA-4, PD-1, IL-1, IL-2, IL-4, IL-10, IL-17, IFN-y, TGF-B and PD-L1
Kaewkangsadan 2017 [14]	TIMs (M1-M2), TINs, DCs
Ladoire 2008 [15]	CD3, CD8, FOXP3 and CD8/FOXP3 ratio
Ladoire 2011 [16]	CD8, FOXP3 and CD8/FOXP3 ratio
Lee 2019 [17]	NLR, TILs, CD4+, CD8+, FOXP3+ and PD-L1+
Li 2019 [18]	TILs and IL8
Liang 2021 [19]	TMB, DC50, CD3, CD8, CD4, PD1, TIM3 and CD4/CD8 ratio
Miyashita 2015 [20]	CD8, FOXP3 and CD8/FOXP3 ratio
Nadin 2014 [21]	TILs
Naofumi Oda 2012 [22]	CD8, FoxP3 and IL-17
Pelekanou 2018 [23]	TILs and PD-L1
Sarradin 2021 [24]	TILs, TIM3, LAG3 and PD-L1
Urueña, 2022 [25]	Stromal and intratumoral TILs, CD45, CD3, CD4, CD8, CD20, CD68 and FoxP3.
Vanguri 2022 [26]	Stromal TILs, CD3, CD8, CD68 and FOXP3
Varadan 2016 [27]	Immune index (141 genes)
Verma 2015 [28]	TILs, CD56, IL-2, IFNg and TGFb

Waks 2019 [29]	TILs, CD8. Foxp3, M1, M2, DCs
Wang 2018 [30]	CD8, PD-1, PD-L1, LAG-3
Wesolowski 2020 [31]	TILs, CD8, PD-1 and PD-L1
Zhang 2019 [32]	FoxP3, CD25 and PD-L1

[1] Abdel-Fatah TM, McArdle SE, Johnson C, Moseley PM, Ball GR, Pockley AG, et al. HAGE (DDX43) is a biomarker for poor prognosis and a predictor of chemotherapy response in breast cancer. British journal of cancer. 2014;110(10):2450-61.

[2] Abdelrahman AE, Rashed HE, MostafaToam, Omar A, Abdelhamid MI, Matar I. Clinicopathological significance of the immunologic signature (PDL1, FOXP3+ Tregs, TILs) in early stage triple-negative breast cancer treated with neoadjuvant chemotherapy. Annals of diagnostic pathology. 2021;51:151676.

[3] Alhesa A, Awad H, Bloukh S, Al-Balas M, El-Sadoni M, Qattan D, et al. PD-L1 expression in breast invasive ductal carcinoma with incomplete pathological response to neoadjuvant chemotherapy. International journal of immunopathology and pharmacology. 2022;36:3946320221078433.

[4] Chan MS, Chen SF, Felizola SJ, Wang L, Nemoto N, Tamaki K, et al. Correlation of tumor-infiltrative lymphocyte subtypes alteration with neoangiogenesis before and after neoadjuvant chemotherapy treatment in breast cancer patients. The International journal of biological markers. 2014;29(3):e193-203.

[5] Demaria S, Volm MD, Shapiro RL, Yee HT, Oratz R, Formenti SC, et al. Development of tumor-infiltrating lymphocytes in breast cancer after neoadjuvant paclitaxel chemotherapy. Clinical cancer research : an official journal of the American Association for Cancer Research. 2001;7(10):3025-30.

[6] Demir L, Yigit S, Ellidokuz H, Erten C, Somali I, Kucukzeybek Y, et al. Predictive and prognostic factors in locally advanced breast cancer: effect of intratumoral FOXP3+ Tregs. Clinical & experimental metastasis. 2013;30(8):1047-62.

[7] Dieci MV, Tsvetkova V, Griguolo G, Miglietta F, Tasca G, Giorgi CA, et al. Integration of tumour infiltrating lymphocytes, programmed cell-death ligand-1, CD8 and FOXP3 in prognostic models for triple-negative breast cancer: Analysis of 244 stage I-III patients treated with standard therapy. European journal of cancer. 2020;136:7-15.

[8] Garcia-Martinez E, Gil GL, Benito AC, Gonzalez-Billalabeitia E, Conesa MA, Garcia Garcia T, et al. Tumor-infiltrating immune cell profiles and their change after neoadjuvant chemotherapy predict response and prognosis of breast cancer. Breast cancer research : BCR. 2014;16(6):488.

[9] Graeser M, Feuerhake F, Gluz O, Volk V, Hauptmann M, Jozwiak K, et al. Immune cell composition and functional marker dynamics from multiplexed immunohistochemistry to predict response to neoadjuvant chemotherapy in the WSG-ADAPT-TN trial. Journal for immunotherapy of cancer. 2021;9(5).

[10] Hoffmann LG, Sarian LO, Vassallo J, de Paiva Silva GR, Ramalho SOB, Ferracini AC, et al. Evaluation of PD-L1 and tumor infiltrating lymphocytes in paired pretreatment biopsies and post neoadjuvant chemotherapy surgical specimens of breast carcinoma. Scientific reports. 2021;11(1):22478.

[11] Park YH, Lal S, Lee JE, Choi YL, Wen J, Ram S, et al. Chemotherapy induces dynamic immune responses in breast cancers that impact treatment outcome. Nature communications. 2020;11(1):6175.

[12] Hornychova H, Melichar B, Tomsova M, Mergancova J, Urminska H, Ryska A. Tumor-infiltrating lymphocytes predict response to neoadjuvant chemotherapy in patients with breast carcinoma. Cancer investigation. 2008;26(10):1024-31.

[13] Kaewkangsadan V, Verma C, Eremin JM, Cowley G, Ilyas M, Eremin O. Crucial Contributions by T Lymphocytes (Effector, Regulatory, and Checkpoint Inhibitor) and Cytokines (TH1, TH2, and TH17) to a Pathological Complete Response Induced by Neoadjuvant Chemotherapy in Women with Breast Cancer. Journal of immunology research. 2016;2016:4757405.

[14] Kaewkangsadan V, Verma C, Eremin JM, Cowley G, Ilyas M, Satthaporn S, et al. The Differential Contribution of the Innate Immune System to a Good Pathological Response in the Breast and Axillary Lymph Nodes Induced by Neoadjuvant Chemotherapy in Women with Large and Locally Advanced Breast Cancers. Journal of immunology research. 2017;2017:1049023.
[15] Ladoire S, Arnould L, Apetoh L, Coudert B, Martin F, Chauffert B, et al. Pathologic complete response to neoadjuvant chemotherapy of breast carcinoma is associated with the disappearance of tumor-infiltrating foxp3+ regulatory T cells. Clinical cancer research : an official journal of the American Association for Cancer Research. 2008;14(8):2413-20.
[16] Ladoire S, Mignot G, Dabakuyo S, Arnould L, Apetoh L, Rebe C, et al. In situ immune response after neoadjuvant chemotherapy for breast cancer predicts survival. The Journal of pathology. 2011;224(3):389-400.
[17] Lee J, Kim DM, Lee A. Prognostic Role and Clinical Association of Tumor-Infiltrating Lymphocyte, Programmed Death Ligand-1 Expression with Neutrophil-Lymphocyte Ratio in Locally Advanced Triple-Negative Breast Cancer. Cancer research and treatment. 2019;51(2):649-63.
[18] Li X, Warren S, Pelekanou V, Wali V, Cesano A, Liu M, et al. Immune profiling of pre- and post-treatment breast cancer tissues from the SWOG S0800 neoadjuvant trial. Journal for immunotherapy of cancer. 2019;7(1):88.
[19] Liang H, Huang J, Ao X, Guo W, Chen Y, Lu D, et al. TMB and TCR Are Correlated Indicators Predictive of the Efficacy of Neoadjuvant Chemotherapy in Breast Cancer. Frontiers in oncology. 2021;11:740427.
[20] Miyashita M, Sasano H, Tamaki K, Hirakawa H, Takahashi Y, Nakagawa S, et al. Prognostic significance of tumor-infiltrating CD8+ and FOXP3+ lymphocytes in residual tumors and alterations in these parameters after neoadjuvant chemotherapy in triple-negative breast cancer: a retrospective multicenter study. Breast cancer research : BCR. 2015;17:124.
[21] Nadin SB, Sottile ML, Montt-Guevara MM, Gauna GV, Daguerre P, Leuzzi M, et al. Prognostic implication of HSPA (HSP70) in breast cancer patients treated with neoadjuvant anthracycline-based chemotherapy. Cell stress & chaperones. 2014;19(4):493-505.
[22] Oda N, Shimazu K, Naoi Y, Morimoto K, Shimomura A, Shimoda M, et al. Intratumoral regulatory T cells as an independent predictive factor for pathological complete response to neoadjuvant paclitaxel followed by 5-FU/epirubicin/cyclophosphamide in breast cancer patients. Breast cancer research and treatment. 2012;136(1):107-16.
[23] Pelekanou V, Barlow WE, Nahleh ZA, Wasserman B, Lo YC, von Wahlde MK, et al. Tumor-Infiltrating Lymphocytes and PD-L1 Expression in Pre- and Posttreatment Breast Cancers in the SWOG S0800 Phase II Neoadjuvant Chemotherapy Trial. Molecular cancer therapeutics. 2018;17(6):1324-31.
[24] Sarradin V, Lusque A, Filleron T, Dalenc F, Franchet C. Immune microenvironment changes induced by neoadjuvant chemotherapy in triple-negative breast cancers: the MIMOSA-1 study. Breast cancer research : BCR. 2021;23(1):61.
[25] Uruena C, Lasso P, Bernal-Estevez D, Rubio D, Salazar AJ, Olaya M, et al. The breast cancer immune microenvironment is modified by neoadjuvant chemotherapy. Scientific reports. 2022;12(1):7981.
[26] Vanguri RS, Fenn KM, Kearney MR, Wang Q, Guo H, Marks DK, et al. Tumor Immune Microenvironment and Response to Neoadjuvant Chemotherapy in Hormone Receptor/HER2+ Early Stage Breast Cancer. Clinical breast cancer. 2022;22(6):538-46.
[27] Varadan V, Gilmore H, Miskimen KL, Tuck D, Parsai S, Awadallah A, et al. Immune Signatures Following Single Dose Trastuzumab Predict Pathologic Response to PreoperativeTrastuzumab and Chemotherapy in HER2-Positive Early Breast Cancer. Clinical cancer research : an official journal of the American Association for Cancer Research. 2016;22(13):3249-59.
[28] Verma C, Kaewkangsadan V, Eremin JM, Cowley GP, Ilyas M, El-Sheemy MA, et al. Natural killer (NK) cell profiles in blood and tumour in women with large and locally advanced breast cancer (LLABC) and their contribution to a pathological complete response (PCR) in the tumour following neoadjuvant chemotherapy (NAC): differential restoration of blood profiles by NAC and surgery. Journal of translational medicine. 2015;13:180.
[29] Waks AG, Stover DG, Guerriero JL, Dillon D, Barry WT, Gjini E, et al. The Immune Microenvironment in Hormone Receptor-Positive Breast Cancer Before and After Preoperative

Chemotherapy. Clinical cancer research : an official journal of the American Association for Cancer Research. 2019;25(15):4644-55.
[30] Wang Y, Dong T, Xuan Q, Zhao H, Qin L, Zhang Q. Lymphocyte-Activation Gene-3 Expression and Prognostic Value in Neoadjuvant-Treated Triple-Negative Breast Cancer. Journal of breast cancer. 2018;21(2):124-33.
[31] Wesolowski R, Stiff A, Quiroga D, McQuinn C, Li Z, Nitta H, et al. Exploratory analysis of immune checkpoint receptor expression by circulating T cells and tumor specimens in patients receiving neo-adjuvant chemotherapy for operable breast cancer. BMC cancer. 2020;20(1):445.
[32] Zhang L, Wang XI, Ding J, Sun Q, Zhang S. The predictive and prognostic value of Foxp3+/CD25+ regulatory T cells and PD-L1 expression in triple negative breast cancer. Annals of diagnostic pathology. 2019;40:143-51.
