# Supplementary material for: Effect of neoadjuvant chemotherapy on tumor immune infiltration in breast cancer patients: Systematic review and meta-analysis
Source: PLoS One. 2023 Apr 27;18(4):e0277714. doi: 10.1371/journal.pone.0277714 (PMC10138237; doi:10.1371/journal.pone.0277714)
Supplement: S2 Table — (PDF) [file pone.0277714.s006.pdf]

SUPPLEMENTARY MATERIAL 2

EXCLUDED ARTICLES			
Study			Reason of exclusion
Miskad	2021	(1)	Results do not apply
Cabioglu	2021	(2)	Results do not apply
Deng	2021	(3)	Mixed neoadjuvancy
Da Silva	2021	(4)	Results do not apply
Karn	2020	(5)	Mixed neoadjuvancy
Bacinschi	2020	(6)	Review
Dieci	2020	(7)	Review
Razis	2020	(8)	Patients did not receive NAC as first line of treatment
Koletsa	2020	(9)	Patients did not receive NAC as first line of treatment
Guo	2020	(10)	Results do not apply
Simon	2020	(11)	Results do not apply
Van Bockstal	2020	(12)	Results do not apply
Lundgren	2020	(13)	Results do not apply
Bai	2020	(14)	Results do not apply
Ohno	2020	(15)	Results do not apply
Nome	2020	(16)	Results do not apply
Lander	2020	(17)	Poster
Chumsri	2020	(18)	Poster
Garber	2020	(19)	Poster
Naoko Itoi	2019	(20)	Mixed neoadjuvancy
Cerbelli	2019	(21)	Results do not apply
Luengo-Gil	2019	(22)	Results do not apply
Hida	2019	(23)	Results do not apply
Oliveira	2019	(24)	Results do not apply
Sinn	2019	(25)	Results do not apply
Van Berckelaer	2019	(26)	Results do not apply
Jongen	2019	(27)	Results do not apply
Sharma	2019	(28)	Results do not apply
Noske	2019	(29)	Results do not apply
Zhao	2019	(30)	Results do not apply
Groot	2019	(31)	Results do not apply
Parkes	2019	(32)	Poster
Dieci	2019	(33)	Poster
Nebhan	2019	(34)	Poster
Gluz	2019	(35)	Poster
Passildas-Jahanmohan	2019	(36)	Poster
Kourea	2019	(37)	Poster
Jongen	2019	(38)	Poster
Van Berckelaer	2019	(39)	Poster
Hinerfeld	2019	(40)	Poster
Jongen, L.	2019	(41)	Results do not apply
Force	2018	(42)	Mixed neoadjuvancy
Liang	2018	(43)	Mixed neoadjuvancy
Muntasell	2018	(44)	Mixed neoadjuvancy
Meng	2018	(45)	Patients did not receive NAC as first line of treatment
Li	2018	(46)	Patients did not receive NAC as first line of treatment

Buisseret	2018	(47)	Patients did not receive NAC as first line of treatment
Saraiva	2018	(48)	Results do not apply
Foukakis	2018	(49)	Results do not apply
Ohara	2018	(50)	Results do not apply
Li	2018	(51)	Results do not apply
Kochi	2018	(52)	Results do not apply
Verovkina	2018	(53)	Poster
Li	2018	(54)	Poster
Van Rossum	2018	(55)	Poster
Noske	2018	(56)	Poster
Shim	2018	(57)	Results do not apply
Sinn	2018	(58)	Poster
McLemore	2017	(59)	Not available
Blok	2017	(60)	Results do not apply
Igari	2017	(61)	Results do not apply
Chen	2017	(62)	Results do not apply
Bense	2017	(63)	Results do not apply
Mittendorf	2017	(64)	Poster
Sobral-Leite	2017	(65)	Poster
Perou	2017	(66)	Poster
Liu	2017	(67)	Poster
Roche	2017	(68)	Poster
Sobral-Leite	2017	(69)	Poster
Linnaus	2017	(70)	Poster
Leon-Ferre	2017	(71)	Poster
Rao	2017	(72)	Mixed neoadjuvancy
Brodsky	2016	(73)	Patients did not receive NAC as first line of treatment
Kotoula	2016	(74)	Results do not apply
Hamy	2016	(75)	Results do not apply
Bottai	2016	(76)	Results do not apply
Song	2016	(77)	Results do not apply
Mitrofanova	2016	(78)	Results do not apply
Raphael	2016	(79)	Results do not apply
Asano	2016	(80)	Results do not apply
Lehmann	2016	(81)	Poster
Moyer	2016	(82)	Studies in mice or cell lines
Stoll	2015	(83)	Not available
Chao	2015	(84)	Poster
Bianchini	2015	(85)	Patients did not receive NAC as first line of treatment
Lee	2015	(86)	Results do not apply
Yu	2014	(87)	Results do not apply
Ali	2014	(88)	Results do not apply
Brown	2014	(89)	Results do not apply
Miyashita	2014	(90)	Mixed neoadjuvancy
Seo	2013	(91)	Results do not apply
Schmidt	2012	(92)	Results do not apply
Ruffell	2012	(93)	Results do not apply
Yamaguchi	2012	(94)	Poster
Garcia-Martinez	2012	(95)	Results do not apply
West	2011	(96)	Poster
DeNardo	2011	(97)	Results do not apply

Denkert	2010	(98)	Results do not apply
Rody	2009	(99)	Results do not apply
Bonnefoi	2003	(100)	Results do not apply

References of the excluded articles:

1. Miskad U, Rifai R, Masadah R, Nelwan B, Ahmad D, Cangara H, et al. The value of tumor-infiltrating lymphocytes and CD8 expression as a predictor of response to anthracycline-based neoadjuvant chemotherapy in invasive breast carcinoma of no special type. Breast disease. 2021;40(S1).
2. Cabioglu N, Onder S, Oner G, Karatay H, Tukenmez M, Muslumanoglu M, et al. TIM3 expression on TILs is associated with poor response to neoadjuvant chemotherapy in patients with locally advanced triple-negative breast cancer. BMC cancer. 2021;21(1).
3. Deng J, Thennavan A, Shah S, Bagdatlioglu E, Klar N, Heguy A, et al. Serial single-cell profiling analysis of metastatic TNBC during Nab-paclitaxel and pembrolizumab treatment. Breast cancer research and treatment. 2021;185(1).
4. da Silva J, de Albuquerque L, Rodrigues F, de Mesquita G, Fernandes P, Thuler L, et al. Prognostic Influence of Residual Tumor-Infiltrating Lymphocyte Subtype After Neoadjuvant Chemotherapy in Triple-Negative Breast Cancer. Frontiers in oncology. 2021;11.
5. Karn T, Meissner T, Weber K, Solbach C, Denkert C, Engels K, et al. A Small Hypoxia Signature Predicted pCR Response to Bevacizumab in the Neoadjuvant GeparQuinto Breast Cancer Trial. Clinical cancer research : an official journal of the American Association for Cancer Research. 2020;26(8).
6. Bacinschi X, Zgura A, Safta I, Anghel R. Biomolecular Factors Represented by Bcl-2, p53, and Tumor-Infiltrating Lymphocytes Predict Response for Adjuvant Anthracycline Chemotherapy in Patients with Early Triple-Negative Breast Cancer. Cancer management and research. 2020;12.
7. Dieci M, Arnedos M, Delaloge S, Andre F. Quantification of residual risk of relapse in breast cancer patients optimally treated. Breast (Edinburgh, Scotland). 2013;22 Suppl 2.
8. Razis E, Kalogeras K, Kotsantis I, Koliou G, Manousou K, Wirtz R, et al. The Role of CXCL13 and CXCL9 in Early Breast Cancer. Clinical breast cancer. 2020;20(1).
9. Koletsa T, Kotoula V, Koliou G, Manousou K, Chrisafi S, Zagouri F, et al. Prognostic impact of stromal and intratumoral CD3, CD8 and FOXP3 in adjuvantly treated breast cancer: do they add information over stromal tumor-infiltrating lymphocyte density? Cancer immunology, immunotherapy : CII. 2020;69(8).
10. Guo H, Ding Q, Gong Y, Gilcrease M, Zhao M, Zhao J, et al. Comparison of three scoring methods using the FDA-approved 22C3 immunohistochemistry assay to evaluate PD-L1 expression in breast cancer and their association with clinicopathologic factors. Breast cancer research : BCR. 2020;22(1).
11. Simon V, Laot L, Laas E, Rozette S, Guerin J, Balezeau T, et al. No Impact of Smoking Status on Breast Cancer Tumor Infiltrating Lymphocytes, Response to Neoadjuvant Chemotherapy and Prognosis. Cancers. 2020;12(10).
12. Van Bockstal M, Noel F, Guiot Y, Duhoux F, Mazzeo F, Van Marcke C, et al. Predictive markers for pathological complete response after neo-adjuvant chemotherapy in triple-negative breast cancer. Annals of diagnostic pathology. 2020;49.
13. Lundgren C, Bendahl P, Ekholm M, Fernö M, Forsare C, Krüger U, et al. Tumour-infiltrating lymphocytes as a prognostic and tamoxifen predictive marker in premenopausal breast cancer: data from a randomised trial with long-term follow-up. Breast cancer research : BCR. 2020;22(1).

14. Bai Y, Gao G, Zhang H, Zhang S, Liu Y, Duan X, et al. Prognostic value of tumor-infiltrating lymphocyte subtypes in residual tumors of patients with triple-negative breast cancer after neoadjuvant chemotherapy. Chinese medical journal. 2020;133(5).
15. Ohno S, Saji S, Masuda N, Tsuda H, Akiyama F, Kurosumi M, et al. Relationships between pathological factors and long-term outcomes in patients enrolled in two prospective randomized controlled trials comparing the efficacy of oral tegafur-uracil with CMF (N·SAS-BC 01 trial and CUBC trial). Breast cancer research and treatment. 2021;186(1).
16. Nome M, Euceda L, Jabeen S, Debik J, Bathen T, Giskeødegård G, et al. Serum levels of inflammation-related markers and metabolites predict response to neoadjuvant chemotherapy with and without bevacizumab in breast cancers. International journal of cancer. 2020;146(1).
17. Lander E, Lehmann B, Shah P, Dees E, Ballinger T, Pohlmann P, et al. A phase II trial of atezolizumab (anti-PD-L1) with carboplatin in patients with metastatic triple-negative breast cancer (mTNBC). Journal of Clinical Oncology. 2020.
18. Chumsri S, Carter J, Ma Y, Hinerfeld D, Brauer H, Warren S, et al. Role of intratumoral NK cells in triple-negative breast cancer in the FinXX trial and Mayo Clinic cohort. Journal of Clinical Oncology. 2020.
19. Garber H, Rauch G, Adrada B, Candelaria R, Mittendorf E, Thompson A, et al. Residual cancer burden in patients with early stage triple negative breast cancer who progress on anthracycline-based neoadjuvant chemotherapy in an ongoing clinical trial (ARTEMIS). Cancer Research. 2020.
20. Itoi N, Umeda T, Ishida M, Murata S, Mori T, Kawai Y, et al. Infiltration of CD4, CD8, CD56, and Fox-P3-positive lymphocytes in breast carcinoma tissue after neoadjuvant chemotherapy with or without trastuzumab. Breast disease. 2019;38(2).
21. Cerbelli B, Botticelli A, Pisano A, Pernazza A, Campagna D, De Luca A, et al. CD73 expression and pathologic response to neoadjuvant chemotherapy in triple negative breast cancer. Virchows Archiv : an international journal of pathology. 2020;476(4).
22. Luengo-Gil G, García-Martínez E, Chaves-Benito A, Conesa-Zamora P, Navarro-Manzano E, González-Billalabeitia E, et al. Clinical and biological impact of miR-18a expression in breast cancer after neoadjuvant chemotherapy. Cellular oncology (Dordrecht). 2019;42(5).
23. Hida A, Watanabe T, Sagara Y, Kashiwaba M, Sagara Y, Aogi K, et al. Diffuse distribution of tumor-infiltrating lymphocytes is a marker for better prognosis and chemotherapeutic effect in triple-negative breast cancer. Breast cancer research and treatment. 2019;178(2).
24. Oliveira M, Saura C, Nuciforo P, Calvo I, Andersen J, Passos-Coelho J, et al. FAIRLANE, a double-blind placebo-controlled randomized phase II trial of neoadjuvant ipatasertib plus paclitaxel for early triple-negative breast cancer. Annals of oncology : official journal of the European Society for Medical Oncology. 2019;30(8).
25. Sinn B, Weber K, Schmitt W, Fasching P, Symmans W, Blohmer J, et al. Human leucocyte antigen class I in hormone receptor-positive, HER2-negative breast cancer: association with response and survival after neoadjuvant chemotherapy. Breast cancer research : BCR. 2019;21(1).
26. Van Berckelaer C, Rypens C, van Dam P, Pouillon L, Parizel M, Schats K, et al. Infiltrating stromal immune cells in inflammatory breast cancer are associated with an improved outcome and increased PD-L1 expression. Breast cancer research : BCR. 2019;21(1).
27. Jongen L, Floris G, Laenen A, Ardui J, .Vergote I, Berteloot P, et al. The prognostic and predictive role of the androgen receptor in TNBC treated with neo-adjuvant chemotherapy. Annals of Oncology. 2019;30.
28. Sharma P, Barlow W, Godwin A, Parkes E, Knight L, Walker S, et al. Validation of the DNA Damage Immune Response Signature in Patients With Triple-Negative Breast Cancer From the SWOG 9313c Trial. Journal of clinical oncology : official journal of the American Society of Clinical Oncology. 2019;37(36).

29. Noske A, Möbus V, Weber K, Schmatloch S, Weichert W, Köhne C, et al. Relevance of tumour-infiltrating lymphocytes, PD-1 and PD-L1 in patients with high-risk, nodal-metastasised breast cancer of the German Adjuvant Intergroup Node-positive study. European journal of cancer (Oxford, England : 1990). 2019;114.
30. Zhao C, Singh K, Brodsky A, Lu S, Graves T, Fenton M, et al. Stromal ColXα1 expression correlates with tumor-infiltrating lymphocytes and predicts adjuvant therapy outcome in ER-positive/HER2-positive breast cancer. BMC cancer. 2019;19(1).
31. de Groot A, Blok E, Charehbili A, Engels C, Smit V, Dekker-Ensink N, et al. Strong CD8+ lymphocyte infiltration in combination with expression of HLA class I is associated with better tumor control in breast cancer patients treated with neoadjuvant chemotherapy. Breast cancer research and treatment. 2019;175(3).
32. Parkes E, James C, Lioe T, Walker S, Savage K, Lowry K, et al. Biomarker phase neo-DDRD trial: Predicting response to neoadjuvant chemotherapy (NAC) in early breast cancer using the DNA damage repair deficiency (DDRD) assay | Cancer Research | American Association for Cancer Research. Cancer Research. 2019.
33. Dieci M, Tsvetkova V, Griguolo G, Miglietta F, Bacchin D, Tasca G, et al. Integrating CD8, FOXP3 and PD-L1 expression in prognostic models for triple negative breast cancer (TNBC): An analysis of 265 patients treated with standard therapy for stage I-III disease | Cancer Research | American Association for Cancer Research. Cancer Research. 2019.
34. Nebhan C, Gonzalez-Ericsson P, Salgado R, Bordeaux J, Young Kim J, Vaupel C, et al. Molecular characterization of residual triple-negative breast cancers after neoadjuvant chemotherapy identifies immune composition and features associated with clinical outcome | Cancer Research | American Association for Cancer Research. Cancer Research. 2019.
35. Gluz O, Nitz U, Liedtke C, Prat A, Christgen M, Feuerhake M, et al. No survival benefit of chemotherapy escalation in patients with pCR and “high-immune” triple-negative early breast cancer in the neoadjuvant WSG-ADAPT-TN trial | Cancer Research | American Association for Cancer Research. 2019.
36. Passildas-Jahanmohan J, Ouled Dhaou M, Kwiatkowski F, Finck W, Poirier C, Mouret-Reynier M, et al. Pattern and biomarkers of recurrence in 305 triple negative breast cancer patients treated in a French comprehensive cancer center | Cancer Research | American Association for Cancer Research. Cancer Research. 2019.
37. Kourea H, Koletsa T, Kotoula V, Koliou G-A, Batistatou A, Pentheroudakis G, et al. Prognostic significance of CD8+ tumor-infiltrating lymphocytes (TILs) in patients with early breast cancer (EBC) treated with dose-dense sequential adjuvant chemotherapy (dds-CT). An observational study (ACTRN12616001043426) | Cancer Research | American Association for Cancer Research. Cancer Research. 2019.
38. Jongen L, Floris G, Wildiers H, Claessens F, De Sutter L, Croes R, et al. The prognostic role of the androgen receptor in patients with triple-negative early breast cancers and primary surgery. 2019.
39. Van Berckelaer C, Colpaert C, Rypens C, Marien K, Waumans Y, Kockx M, et al. Abstract P4-06-08: The spatial localization of immune cells predicts prognosis and response to therapy in inflammatory breast cancer | Cancer Research | American Association for Cancer Research. Cancer Research. 2010.
40. Hinerfeld D, Chumsri S, Asleh K, Brauer H, Kachergus J, Lauttia S, et al. Effects of immune architecture on response to adjuvant capecitabine in triple negative breast cancer (FinXX trial). Annals of Oncology. 2019;30.

41. Jongen L, Floris G, Wildiers H, Claessens F, Richard F, Laenen A, et al. Tumor characteristics and outcome by androgen receptor expression in triple-negative breast cancer patients treated with neo-adjuvant chemotherapy. Breast cancer research and treatment. 2019;176(3).
42. Force J, Howie L, Abbott S, Bentley R, Marcom P, Kimmick G, et al. Early Stage HER2-Positive Breast Cancers Not Achieving a pCR From Neoadjuvant Trastuzumab- or Pertuzumab-Based Regimens Have an Immunosuppressive Phenotype. Clinical breast cancer. 2018;18(5).
43. Liang X, Briaux A, Becette V, Benoist C, Boulai A, Chemlali W, et al. Molecular profiling of hormone receptor-positive, HER2-negative breast cancers from patients treated with neoadjuvant endocrine therapy in the CARMINA 02 trial (UCBG-0609). Journal of hematology & oncology. 2018;11(1).
44. Muntasell A, Rojo F, Servitja S, Rubio-Perez C, Cabo M, Tamborero D, et al. NK Cell Infiltrates and HLA Class I Expression in Primary HER2 + Breast Cancer Predict and Uncouple Pathological Response and Disease-free Survival. Clinical cancer research : an official journal of the American Association for Cancer Research. 2019;25(5).
45. Meng S, Li L, Zhou M, Jiang W, Niu H, Yang K. Distribution and prognostic value of tumor-infiltrating T cells in breast cancer. Molecular medicine reports. 2018;18(5).
46. Li Y, Opyrchal M, Yao S, Peng X, Yan L, Jabbour H, et al. The role of programmed death ligand-1 and tumor-infiltrating lymphocytes in breast cancer overexpressing HER2 gene. Breast cancer research and treatment. 2018;170(2).
47. Buisseret L, Pommey S, Allard B, Garaud S, Bergeron M, Cousineau I, et al. Clinical significance of CD73 in triple-negative breast cancer: multiplex analysis of a phase III clinical trial. Annals of oncology : official journal of the European Society for Medical Oncology. 2018;29(4).
48. Saraiva D, Jacinto A, Borralho P, Braga S, Cabral M. HLA-DR in Cytotoxic T Lymphocytes Predicts Breast Cancer Patients' Response to Neoadjuvant Chemotherapy. Frontiers in immunology. 2018;9.
49. Foukakis T, Lövrot J, Matikas A, Zerdes I, Lorent J, Tobin N, et al. Immune gene expression and response to chemotherapy in advanced breast cancer. British Journal of Cancer. 2018;118(4):480-8.
50. Ohara A, Naoi Y, Shimazu K, Kagara N, Shimoda M, Tanei T, et al. PAM50 for prediction of response to neoadjuvant chemotherapy for ER-positive breast cancer. Breast cancer research and treatment. 2019;173(3).
51. Li F, Zhao Y, Wei L, Li S, Liu J. Tumor-infiltrating Treg, MDSC, and IDO expression associated with outcomes of neoadjuvant chemotherapy of breast cancer. Cancer biology & therapy. 2018;19(8).
52. Kochi M, Iwamoto T, Niikura N, Bianchini G, Masuda S, Mizoo T, et al. Tumour-infiltrating lymphocytes (TILs)-related genomic signature predicts chemotherapy response in breast cancer. Breast cancer research and treatment. 2018;167(1).
53. Verovkina N, Lyalkin S, Syvak L, Askolskyi A, Majdanevych N. CD8+, CD4+ and FOXP3+ cell profiles and their change after neoadjuvant chemotherapy in patients with triple negative breast cancer | OncologyPRO. Oncology pro. 2018.
54. Li X, Warren S, Pelekanou V, Wali V, Cesano A, Liu M, et al. Immune profiling of pre- and post-treatment breast cancer tissues from the S0800 randomized neoadjuvant trial of weekly nab-paclitaxel with or without bevacizumab and dose dense doxorubicin and cyclophosphamide. https://doiorg/101200/JCO20183615_suppl578. 2018.
55. van Rossum A, Hoogstraat M, Opdam M, Horlings H, Wessels L, Kerkhoven R, et al. Tumor infiltrating lymphocytes predict benefit from TAC but not from ddAC in triple negative breast cancer in the randomized MATADOR trial (BOOG 2004-04). Annals of Oncology. 2018;29.

56. Noske A, Weber K, Moebus V, Schmatloch S, Weichert W, Kohne C, et al. Tumor infiltrating lymphocytes to predict DFS from intense dose-dense (idd) EPC regimen: Results from the German Adjuvant Intergroup Node-positive study (GAIN-1). https://doiorg/101200/JCO20183615_suppl527. 2018.
57. Shim B, Jin M, Moon J, Park I, Ryu H. High Cytoplasmic CXCR4 Expression Predicts Prolonged Survival in Triple-Negative Breast Cancer Patients Treated with Adjuvant Chemotherapy. Journal of pathology and translational medicine. 2018;52(6).
58. Sinn B, Weber K, Denkert C, Fasching P, Schmitt W, Thomas K, et al. HLA class I expression is associated with tumor-infiltrating lymphocytes and response and survival after neoadjuvant chemotherapy in hormone receptor-positive, HER2-negative breast cancer | Cancer Research | American Association for Cancer Research. Cancer Research. 2018.
59. McLemore L, Janakiram M, Albanese J, Shapiro N, Lo Y, Zang X, et al. An Immunoscore Using PD-L1, CD68, and Tumor-infiltrating Lymphocytes (TILs) to Predict Response to Neoadjuvant Chemotherapy in Invasive Breast Cancer. Applied immunohistochemistry & molecular morphology : AIMM. 2018;26(9).
60. Blok E, van den Bulk J, Dekker-Ensink N, Derr R, Kanters C, Bastiaannet E, et al. Combined evaluation of the FAS cell surface death receptor and CD8+ tumor infiltrating lymphocytes as a prognostic biomarker in breast cancer. Oncotarget. 2017;8(9).
61. Igari F, Sato E, Horimoto Y, Takahashi Y, Isomura T, Arakawa A, et al. Diagnostic significance of intratumoral CD8+ tumor-infiltrating lymphocytes in medullary carcinoma. Human pathology. 2017;70.
62. Chen S, Wang R, Liu Y, Yang W, Shao Z. PD-L1 expression of the residual tumor serves as a prognostic marker in local advanced breast cancer after neoadjuvant chemotherapy. International journal of cancer. 2017;140(6).
63. Bense R, Sotiriou C, Piccart-Gebhart M, Haanen J, van Vugt M, de Vries E, et al. Relevance of Tumor-Infiltrating Immune Cell Composition and Functionality for Disease Outcome in Breast Cancer. Journal of the National Cancer Institute. 2016;109(1).
64. Mittendorf E, Barrios C, Harbeck N, Jung K, Miles D, Saji S, et al. IMpassion031: A phase III study comparing neoadjuvant atezolizumab (atezo) vs placebo in combination with anthracycline/nab-paclitaxel (nab-pac)–based chemotherapy in early triple-negative breast cancer (eTNBC). Annals of Oncology. 2017;28.
65. Sobral-Leite M, Van de Vijver K, Michaut M, Horlings H, Severson T, Schouten P, et al. PD-L1 positive tumor-infiltrating lymphocytes and mutational load in breast cancer | Cancer Research | American Association for Cancer Research. Cancer Research. 2017.
66. Perou C. Precision medicine for triple-negative breast cancer patients using a systems biology approach | Molecular Cancer Research | American Association for Cancer Research. Molecular cancer research. 2017.
67. Liu S, Chen B, Burugu S, Leung S, Gao D, Virk S, et al. Predictive effect of cytotoxic tumor infiltrating lymphocytes in HER2-positive metastatic breast cancer: A correlative study with CCTG MA.31 | Cancer Research | American Association for Cancer Research. Cancer Research. 2017.
68. Roché H, Lafouresse F, Filleron T, Laffont R, Maisongrosse V, Pichery M, et al. Prognostic and predictive values of high endothelial venules (HEV) and tumor infiltrating CD8+ lymphocytes (CD8) in tumors of patients included in the adjuvant PACS04 trial: HEV is predictive of outcome for HER2+ tumors exposed to trastuzumab | Cancer Research | American Association for Cancer Research. Cancer Research. 2017.
69. Sobral-Leite M, Salomon I, Opdam M, Beelen K, van Vlierberghe R, Blok E, et al. Cancer-immune interactions in luminal breast cancers: PI3KCA mutations, PI3K/AKT/mTOR activation and

tumor-infiltrating lymphocytes | Cancer Research | American Association for Cancer Research. Cancer Research. 2017.
70. Linnaus M, Kosiorek H, Barrett M, Dueck A, Anderson K, Ocal I, et al. Effect of PD-1 and PD-L1 in the tumor microenvironment on overall survival of triple-negative breast cancer patients | Cancer Research | American Association for Cancer Research. Cancer Research. 2017.
71. Leon-Ferre L, Polley M, Liu H, Gilbert J, Cafourek V, Hillman D, et al. Prognostic value of histopathology, stromal tumor infiltrating lymphocytes (sTILs) and adjuvant chemotherapy (AdjCT) in early stage triple negative breast cancer (TNBC). https://doiorg/101200/JCO20173515_suppl533. 2017.
72. Rao N, Qiu J, Wu J, Zeng H, Su F, Qiu K, et al. Significance of Tumor-Infiltrating Lymphocytes and the Expression of Topoisomerase IIα in the Prediction of the Clinical Outcome of Patients with Triple-Negative Breast Cancer after Taxane-Anthracycline-Based Neoadjuvant Chemotherapy. Chemotherapy. 2017;62(4).
73. Brodsky A, Xiong J, Yang D, Schorl C, Fenton M, Graves T, et al. Identification of stromal ColXα1 and tumor-infiltrating lymphocytes as putative predictive markers of neoadjuvant therapy in estrogen receptor-positive/HER2-positive breast cancer. BMC cancer. 2016;16.
74. Kotoula V, Karavasilis V, Zagouri F, Kouvatseas G, Giannoulatou E, Gogas H, et al. Effects of TP53 and PIK3CA mutations in early breast cancer: a matter of co-mutation and tumor-infiltrating lymphocytes. Breast cancer research and treatment. 2016;158(2).
75. Hamy A, Bonsang-Kitzis H, Lae M, Moarii M, Sadacca B, Pinheiro A, et al. A Stromal Immune Module Correlated with the Response to Neoadjuvant Chemotherapy, Prognosis and Lymphocyte Infiltration in HER2-Positive Breast Carcinoma Is Inversely Correlated with Hormonal Pathways. PloS one. 2016;11(12).
76. Bottai G, Raschioni C, Losurdo A, Di Tommaso L, Tinterri C, Torrisi R, et al. An immune stratification reveals a subset of PD-1/LAG-3 double-positive triple-negative breast cancers. Breast cancer research : BCR. 2016;18(1).
77. Song I, Heo S, Bang W, Park H, Park I, Kim Y, et al. Predictive Value of Tertiary Lymphoid Structures Assessed by High Endothelial Venule Counts in the Neoadjuvant Setting of Triple-Negative Breast Cancer. Cancer research and treatment. 2017;49(2).
78. Mitrofanova I, Zavyalova M, Telegina N, Buldakov M, Riabov V, Cherdyntseva N, et al. Tumor-associated macrophages in human breast cancer parenchyma negatively correlate with lymphatic metastasis after neoadjuvant chemotherapy. Immunobiology. 2016;222(1).
79. Raphael J, Gong I, Nofech-Mozes S, Bartlett J, Nafisi H, Verma S. Tumour infiltrating lymphocytes and stromal CD68 in early stage HER2 positive breast cancer. Journal of clinical pathology. 2016;69(6).
80. Asano Y, Kashiwagi S, Goto W, Kurata K, Noda S, Takashima T, et al. Tumour-infiltrating CD8 to FOXP3 lymphocyte ratio in predicting treatment responses to neoadjuvant chemotherapy of aggressive breast cancer. The British journal of surgery. 2016;103(7).
81. Lehmann B, Jovanović B, Chen X, Estrada M, Johnson K, Shyr Y, et al. Refinement of Triple-Negative Breast Cancer Molecular Subtypes: Implications for Neoadjuvant Chemotherapy Selection. PloS one. 2016;11(6).
82. Moyer A, Boughey J, Kalari K, Suman V, McLaughlin S, Moreno-Aspitia A, et al. Differential mRNA expression patterns in breast tumors with high vs. low quantity of stromal tumor–Infiltrating lymphocytes | Cancer Research | American Association for Cancer Research. 2016.
83. Stoll G, Bindea G, Mlecnik B, Galon J, Zitvogel L, Kroemer G. Meta-analysis of organ-specific differences in the structure of the immune infiltrate in major malignancies. Oncotarget. 2015;6(14).

84. Chao N, Ting Z, Yun C, Jingxia Z, Tao Z, Jian H, et al. CD73+ gd2 T cells is the dominant immune suppressive cells in breast cancer and correlate with the tumor burden. European Journal of Cancer. 2015;51:S18.
85. Bianchini G, Pusztai L, Pienkowski T, Im Y, Bianchi G, LM T, et al. Immune modulation of pathologic complete response after neoadjuvant HER2-directed therapies in the NeoSphere trial. Annals of oncology : official journal of the European Society for Medical Oncology. 2015;26(12).
86. Lee H, Lee J, Song I, Park I, Kang J, Yu J, et al. Prognostic and predictive value of NanoString-based immune-related gene signatures in a neoadjuvant setting of triple-negative breast cancer: relationship to tumor-infiltrating lymphocytes. Breast cancer research and treatment. 2015;151(3).
87. Yu H, Yang J, Jiao S, Wang J, Li Y. TGF-β1 precursor and CD8 are potential prognostic and predictive markers in operated breast cancer. Journal of Huazhong University of Science and Technology Medical sciences = Hua zhong ke ji da xue xue bao Yi xue Ying De wen ban = Huazhong keji daxue xuebao Yixue Yingdewen ban. 2014;34(1).
88. Ali H, Provenzano E, Dawson S, Blows F, Liu B, Shah M, et al. Association between CD8+ T-cell infiltration and breast cancer survival in 12,439 patients. Annals of oncology : official journal of the European Society for Medical Oncology. 2014;25(8).
89. Brown J, Wimberly H, Lannin D, Nixon C, Rimm D, Bossuyt V. Multiplexed quantitative analysis of CD3, CD8, and CD20 predicts response to neoadjuvant chemotherapy in breast cancer. Clinical cancer research : an official journal of the American Association for Cancer Research. 2014;20(23).
90. Miyashita M, Sasano H, Tamaki K, Chan M, Hirakawa H, Suzuki A, et al. Tumor-infiltrating CD8+ and FOXP3+ lymphocytes in triple-negative breast cancer: its correlation with pathological complete response to neoadjuvant chemotherapy. Breast cancer research and treatment. 2014;148(3).
91. Seo A, Lee H, Kim E, Kim H, Jang M, Lee H, et al. Tumour-infiltrating CD8+ lymphocytes as an independent predictive factor for pathological complete response to primary systemic therapy in breast cancer. British journal of cancer. 2013;109(10).
92. Schmidt M, Hellwig B, Hammad S, Othman A, Lohr M, Chen Z, et al. A comprehensive analysis of human gene expression profiles identifies stromal immunoglobulin κ C as a compatible prognostic marker in human solid tumors. Clinical cancer research : an official journal of the American Association for Cancer Research. 2012;18(9).
93. Ruffell B, Au A, Rugo H, Esserman L, Hwang E, Coussens L. Leukocyte composition of human breast cancer. Proceedings of the National Academy of Sciences of the United States of America. 2012;109(8).
94. Yamaguchi R, Tanaka M, Yano A, Tse G, Yamaguchi M, Koura K, et al. Tumor-infiltrating lymphocytes are important pathologic predictors for neoadjuvant chemotherapy in patients with breast cancer. Human pathology. 2012;43(10).
95. García-Martínez E, Luengo G, Chaves B, García G, Vicente, CAM, Zafra P, et al. Baseline CD4/CD8 tumor infiltrating lymphocytes (TIL) ratio predicts pathologic response to neoadjuvant chemotherapy (NC) in breast cancer | Cancer Research | American Association for Cancer Research. Cancer Research. 2012.
96. West N, Milne K, Truong P, Macpherson N, Nelson B, Watson P. Tumor-infiltrating lymphocytes predict response to anthracycline-based chemotherapy in estrogen receptor-negative breast cancer. Breast cancer research : BCR. 2011;13(6).
97. DeNardo D, Brennan D, Rexhepaj E, Ruffell B, Shiao S, Madden S, et al. Leukocyte complexity predicts breast cancer survival and functionally regulates response to chemotherapy. Cancer discovery. 2011;1(1).

98. Denkert C, Loibl S, Noske A, Roller M, Müller B, Komor M, et al. Tumor-associated lymphocytes as an independent predictor of response to neoadjuvant chemotherapy in breast cancer. Journal of clinical oncology : official journal of the American Society of Clinical Oncology. 2010;28(1).
99. Rody A, Holtrich U, Pusztai L, Liedtke C, Gaetje R, Ruckhaeberle E, et al. T-cell metagene predicts a favorable prognosis in estrogen receptor-negative and HER2-positive breast cancers. Breast cancer research : BCR. 2009;11(2).
100. Bonnefoi H, Diebold-Berger S, Therasse P, Hamilton A, van de Vijver M, MacGrogan G, et al. Locally advanced/inflammatory breast cancers treated with intensive epirubicin-based neoadjuvant chemotherapy: are there molecular markers in the primary tumour that predict for 5-year clinical outcome? Annals of oncology : official journal of the European Society for Medical Oncology. 2003;14(3).
