# Supplementary material for: Effect of neoadjuvant chemotherapy on tumor immune infiltration in breast cancer patients: Systematic review and meta-analysis
Source: PLoS One. 2023 Apr 27;18(4):e0277714. doi: 10.1371/journal.pone.0277714 (PMC10138237; doi:10.1371/journal.pone.0277714)
Supplement: S3 Table — (PDF) [file pone.0277714.s007.pdf]

SUPPLEMENTARY MATERIAL 3

HETEROGENEITY IN TUMOR CLASSIFICATION, TREATMENT RECEIVED AND TUMOR TOPOLOGY AMONG INCLUDED ARTICLES			
**Author and year**	**Type of cancer, or reported diagnosis**	**Reported treatment**	**TME Topology**
Abdel-Fatah 2014 [1]	Primary invasive BC	FEC/FAC 122 (62.3%), FEC+T 19 (9.7%), T-FEC 18 (9.2%), EC+T 14 (7.2%), EC+GT 5 (2.5%), AC-T 15 (7.7%), AC 1 (0.5%), T 2 (1.0%). 63% of patients received six cycles of FEC	Did not differentially evaluate tumor and stroma
Abdelrahman, 2021 [2]	TNBC: T1: 20 (40%), T2: 30 (60%), Node Positive: 32 (64%), Node Negative: 18 (36%) Grade: Grade II:15 (30%), Grade III: 35 (70%)	AC + P	PD-L1 y FoxP3 - Global, Stromal TILs
Alhesa 2022 [3]	Luminal A, Luminal B, HER-2-enriched and TNBC.	55 patients: T + A + C, 5 patients: C + E + F	Stromal
Chan 2014 [4]	Primary breast invasive ductal carcinoma	(i) Canadian CEF: C 75 mg/m2 + E 60 mg/m2 + F 500 mg/m2 6 cycles; or (ii) FEC 100: F 500 mg/m2, E 100 mg/m2 , C 500 mg/m2 4 cycles; with or without subsequent T 75 mg/m2 4 cycles.	Did not differentially evaluate tumor and stroma
Demaria 2001 [5]	Primary BC, T2 or greater with no evidence of systemic metastatic disease. Ductal, well differentiated =1, Ductal, moderately differentiated =5, Ductal, poorly differentiated =15, Ductal, medullary features (atypical medullary) =3 and Lobular =2	P (200 mg/m2) four cycles. Surgery was performed 2–3 weeks after the last dose of paclitaxel.	Did not differentially evaluate tumor and stroma
Demir 2013 [6]	LLABC -Histologic classification: Invasive ductal carcinoma= 85, Invasive lobular carcinoma= 6, Invasive ductal + lobular carcinoma = 4, Invasive papillary carcinoma= 1, Invasive ductal + mucinous carcinoma= 1, Medullary carcinoma= 2, Invasive micropapillary carcinoma= 1, Apocrine carcinoma = 1. Molecular classification: HR(-) HER2(-)= 19, HR(-) HER2(+)= 14, HR(+) HER2(-)= 50, HR(+) HER2(+)= 18. Histological grade (Nottingham Combined Grading system): HG I=7, HG II=66, HG III: 28.	FEC n=26, TEC n=67, ET n=2, FEC + T n= 1, TC n=1, AC + P n=1	Intratumoral
Dieci 2020 [7]]	Non-metastatic TNBC	Patients with TNBC, all treated with surgery and chemotherapy. 86% of patients received anthracycline-containing treatment and 72% received anthracycline/taxane-based treatment.	Stromal
García-Martínez 2014 [8]	Patients with stage II or III BC who received NCT. 63.6% ER+ PR+ (13.2% HER2+, 50.4% HER2-), 10,7% HER2+ (ER- PR-) and 21.5% TNBC.	C (600 mg/m2) and D (60 mg/m2) four courses, followed by T (100 mg/m2) for four cycles.	Intratumoral

Graeser 2021 [9]	Histologically confirmed unilateral, primary invasive cT1c-cT4c or cN+ TNBC	54% patients received nab-P+G, 46% patients received nab-P+CP	Stromal and intratumoral
Grecco-Hoffman 2021 [10]	Women with invasive BC who underwent NACT and post-NACT surgery were included	Luminal BC: 4 cycles of AC + T for 12 cycles. TNBC CP Is added to standard NACT. After NACT all women underwent surgical treatment mastectomy or quadrantectomy with sentinel lymph node biopsy or axillary lymph node dissection.	Stromal
Hee Park 2020 [11]	Histologically confirmed invasive BC: ER+ /HER2−, ER+ /HER2+, HER2+ /ER− and TN	Protocol of 4 cycles of Anthracycline and C followed by four cycles of D, following ASCO Guidelines.	Stromal and intratumoral
Hornychova 2008 [12]	Histologically confirmed BC. 44 patients had stage II disease, 27 patients had stage III disease, and two patients had stage IV disease (limited metastatic disease).	4-6 cycles of the combination of D (50 mg/m2) and P (175 mg/m2) every three weeks.	Stromal and intratumoral
Kaewkangsadan 2016 [13]	LLABC (> 3cm) ER status= 22+ 11- HER-2 status: 10+ 23-	Either 4 courses of AC followed by 4 courses of T ± X or 2 courses of AC followed by 6 courses of T ± X (AC-TX: 16/33, AC-T: 17/33)	Stromal and intratumoral
Kaewkangsadan 2017 [14]	LLABC (> 3cm/T3, 4; N1, 2; M0). ER status= 22+ 11-. HER-2 status: 10+ 23-.	All patients received 8 cycles of NAC, A + C + D + C =16, A + C+ D = 17.	Stromal and intratumoral
Ladoire 2008 [15]	56 patients. Tumoral status: T1 = 1, T2 = 33, T3 = 17, T4 = 5. Node status: N0 = 17, N1 = 31, N2 = 6, N3 = 2. Tumoral grade: I=4, II=33, III=19. Receptor expression: HER2+=20, ER+=37, ER-=19, PR+=27, PR-=29.	FEC 100 (E 100 mg/m2, C 500 mg/m2, and F 500 mg/m2) or CEX (E 100 mg/m2, C 500 mg/m2, and X 900 mg/m2); taxane regimens were used sequentially (with T 100 mg/m2).	Did not differentially evaluate tumor and stroma
Ladoire 2011 [16]	Non-metastatic BC HER2 + and HER2-	FEC 100 (E 100 mg/m2, C 500 mg/m2, and F 500 mg/m2) or FAC (A 50 mg/m2, C 500 mg/m2, and F 500 mg/m2).	Did not differentially evaluate tumor and stroma
Lee 2019 [17]	Locally advanced TNBC stage I to III	Anthracycline and taxane based NAC regimen: (1) T (75 mg/m2) and A (50 mg/m2) for 2-6 cycles; (2) A (60 mg/m2) and C (600 mg/m2) for 4 cycles; (3) E (75 mg/m2) and C (600 mg/m2) for 4 cycles.	Stromal and intratumoral
Li 2019 [18]	HER2-negative, locally advanced, or inflammatory breast cancer	Three arms of the trial were weekly nab-paclitaxel and bevacizumab followed by dose-dense doxorubicin/cyclophosphamide (ddAC) (Arm A), nab-paclitaxel followed by ddAC, (Arm B), and ddAC followed by nab-paclitaxel (Arm C). Patients were randomly allocated in 2:1:1 ratio to arms A, B and C, respectively.	Intratumoral
Liang 2021 [19]	Stage IIB to IIIC BC patients, receiving neoadjuvant chemotherapy (NAC)	TEC or EC followed by T regimen (T: docetaxel/liposome paclitaxel, E: pirarubicin/ epirubicin, C: cyclophosphamide) for 2–8 cycles	Did not differentially evaluate tumor and stroma
Miyashita 2015 [20]	TNBC patients who received NAC American Pathologists guidelines (ER, PgR, HER2, Ki-67, EGFR, and CK5/6)	Of the 131 patients with TNBC, 110 (84 %) received NAC containing both anthracyclines and taxanes as current standard regimens. Nineteen patients received anthracycline-based regimens	Stromal

		for NAC, and the great majority received taxane-based regimens as adjuvant therapy after surgery	
Nadin 2014 [21]	LLABC. Tumoral grade: II = 28 (46.7 %), III = 32 (53.3 %). Size T2 (>20 and ≤50 mm) = 19 (31.7 %), T3 (>50 mm) = 36 (60.0 %) T4 = 5 (8.3 %). Histology: Ductal: 56 patients (93.3 %), Lobular: 4 patients (6.7 %). Metastasis: Metastasis free at the time of diagnosis by careful clinical evaluation, by X-ray of the chest, bone scintigraphy, and liver ultrasound.	4 cycles of 75 mg/m2 D-based nonchemotherapy or 120 mg/m2 E-based nonchemotherapy	Stromal and intratumoral
Naofumi Oda 2012 [22]	Primary invasive BC stage II or III	P (80 mg/m2) 12 cycles + F (500 mg/m2), E (75 mg/m2), C (500 mg/m2) 4 cycles.	Intratumoral
Pelekanou 2018 [23]	HR-positive: ER+ or PR+ (68,7%), HR-negative: ER− and PR− (31,3%). IBC (10,4%), LABC (89,6%)	Three arms of the trial were weekly nab-paclitaxel and bevacizumab followed by dose-dense doxorubicin/cyclophosphamide (ddAC; Arm A), nab-paclitaxel followed by ddAC (Arm B), and ddAC followed by nab-paclitaxel (Arm C)	Stromal
Sarradin 2021 [24]	TNBC	n=1 F (500 mg/m2) + E (100 mg/m2) + C (500 mg/m2). n=36 F (500 mg/m2) + E (100 mg/m2) + C (500 mg/m2) + P (80 mg/m2), n=26 F (500 mg/m2) + E (100 mg/m2) + C (500 mg/m2) + T (100 mg/m2), n=3 F (500 mg/m2) + E (100 mg/m2) + C (500 mg/m2) + T (100 mg/m2) + P (80 mg/m2)	Stromal
Uruena, 2022 [25]	BC	n=4 Anthracyclines + C, n=20 Anthracyclines + C + T	Stromal and intratumoral
Vanguri 2022 [26]	Invasive BC	n=26 Anthracyclines + C, n=14 Anthracyclines + C + T	Stromal
Varadan 2016 [27]	Stage II or III HER2+BC	One dose of nab-P (100 mg/m2) before the posttreatment sample	Did not differentially evaluate tumor and stroma
Verma 2015 [28]	LLABCs (≥3 cm,T3–4, N1–2, M0)	All patients A (60 mg/m2) and C (600 mg/m2). Patients who respond to two courses of AC will be randomized to continue to receive two further courses of AC followed by either four courses of T (100 mg/m2) [Group A] or will receive a combination of T (75 mg/m2) with X (2,000 mg/m2) [Group B]. Nonresponders will be randomized to receive either T (100 mg/m2) for up to six courses [Group C] or a combination of T (75 mg/m2) with X (2,000 mg/m2 [Group D],	Stromal and intratumoral
Waks 2019 [29]	HR+ HER2-	A + C + T all delivered in the neoadjuvant setting	Did not differentially evaluate tumor and stroma
Wang 2018 [30]	Invasive ductal carcinomas	Anthracycline and taxane-based chemotherapy regimens for a median of	Did not differentially

		four cycles (range, three to six cycles) and no other anticancer treatments.	evaluate tumor and stroma
Wesolowski 2020 [31]	n=TNBC, n=8 HR+/HER2- BC, n=3 HR−/HER2+ BC and n=2 HR+/HER2+ BC	dd D (60 mg/m2) and C (600 mg/m2) 4 cycles + P (80 mg/m2) 12 cycles	Stromal and intratumoral
Zhang 2019 [32]	All cases were histologically confirmed as invasive carcinoma of no special type (ductal, not otherwise specified) with an ER/PR/HER2 triple-negative phenotype.	All patients received 4 cycles of doxorubicin + cyclophosphamide followed by 12 cycles of paclitaxel weekly before mastectomy	Did not differentially evaluate tumor and stroma
BC breast cancer, LLABC large and locally advanced breast cancer, IBC inflammatory breast cancer, F 5-fluoracil, E epirubicin, C cyclophosphamide, T docetaxel, P paclitaxel, D doxorubicin, A Adriamycin, G gemcitabine, CP carboplatin, X capecitabine, dd dose dense			

[1] Abdel-Fatah TM, McArdle SE, Johnson C, Moseley PM, Ball GR, Pockley AG, et al. HAGE (DDX43) is a biomarker for poor prognosis and a predictor of chemotherapy response in breast cancer. British journal of cancer. 2014;110(10):2450-61.

[2] Abdelrahman AE, Rashed HE, MostafaToam, Omar A, Abdelhamid MI, Matar I. Clinicopathological significance of the immunologic signature (PDL1, FOXP3+ Tregs, TILs) in early stage triple-negative breast cancer treated with neoadjuvant chemotherapy. Annals of diagnostic pathology. 2021;51:151676.

[3] Alhesa A, Awad H, Bloukh S, Al-Balas M, El-Sadoni M, Qattan D, et al. PD-L1 expression in breast invasive ductal carcinoma with incomplete pathological response to neoadjuvant chemotherapy. International journal of immunopathology and pharmacology. 2022;36:3946320221078433.

[4] Chan MS, Chen SF, Felizola SJ, Wang L, Nemoto N, Tamaki K, et al. Correlation of tumor-infiltrative lymphocyte subtypes alteration with neoangiogenesis before and after neoadjuvant chemotherapy treatment in breast cancer patients. The International journal of biological markers. 2014;29(3):e193-203.

[5] Demaria S, Volm MD, Shapiro RL, Yee HT, Oratz R, Formenti SC, et al. Development of tumor-infiltrating lymphocytes in breast cancer after neoadjuvant paclitaxel chemotherapy. Clinical cancer research : an official journal of the American Association for Cancer Research. 2001;7(10):3025-30.

[6] Demir L, Yigit S, Ellidokuz H, Erten C, Somali I, Kucukzeybek Y, et al. Predictive and prognostic factors in locally advanced breast cancer: effect of intratumoral FOXP3+ Tregs. Clinical & experimental metastasis. 2013;30(8):1047-62.

[7] Dieci MV, Tsvetkova V, Griguolo G, Miglietta F, Tasca G, Giorgi CA, et al. Integration of tumour infiltrating lymphocytes, programmed cell-death ligand-1, CD8 and FOXP3 in prognostic models for triple-negative breast cancer: Analysis of 244 stage I-III patients treated with standard therapy. European journal of cancer. 2020;136:7-15.

[8] Garcia-Martinez E, Gil GL, Benito AC, Gonzalez-Billalabeitia E, Conesa MA, Garcia Garcia T, et al. Tumor-infiltrating immune cell profiles and their change after neoadjuvant chemotherapy predict response and prognosis of breast cancer. Breast cancer research : BCR. 2014;16(6):488.

[9] Graeser M, Feuerhake F, Gluz O, Volk V, Hauptmann M, Jozwiak K, et al. Immune cell composition and functional marker dynamics from multiplexed immunohistochemistry to predict response to neoadjuvant chemotherapy in the WSG-ADAPT-TN trial. Journal for immunotherapy of cancer. 2021;9(5).

[10] Hoffmann LG, Sarian LO, Vassallo J, de Paiva Silva GR, Ramalho SOB, Ferracini AC, et al. Evaluation of PD-L1 and tumor infiltrating lymphocytes in paired pretreatment biopsies and post neoadjuvant chemotherapy surgical specimens of breast carcinoma. Scientific reports. 2021;11(1):22478.

[11] Park YH, Lal S, Lee JE, Choi YL, Wen J, Ram S, et al. Chemotherapy induces dynamic immune responses in breast cancers that impact treatment outcome. Nature communications. 2020;11(1):6175.

[12] Hornychova H, Melichar B, Tomsova M, Mergancova J, Urminska H, Ryska A. Tumor-infiltrating lymphocytes predict response to neoadjuvant chemotherapy in patients with breast carcinoma. Cancer investigation. 2008;26(10):1024-31.
[13] Kaewkangsadan V, Verma C, Eremin JM, Cowley G, Ilyas M, Eremin O. Crucial Contributions by T Lymphocytes (Effector, Regulatory, and Checkpoint Inhibitor) and Cytokines (TH1, TH2, and TH17) to a Pathological Complete Response Induced by Neoadjuvant Chemotherapy in Women with Breast Cancer. Journal of immunology research. 2016;2016:4757405.
[14] Kaewkangsadan V, Verma C, Eremin JM, Cowley G, Ilyas M, Satthaporn S, et al. The Differential Contribution of the Innate Immune System to a Good Pathological Response in the Breast and Axillary Lymph Nodes Induced by Neoadjuvant Chemotherapy in Women with Large and Locally Advanced Breast Cancers. Journal of immunology research. 2017;2017:1049023.
[15] Ladoire S, Arnould L, Apetoh L, Coudert B, Martin F, Chauffert B, et al. Pathologic complete response to neoadjuvant chemotherapy of breast carcinoma is associated with the disappearance of tumor-infiltrating foxp3+ regulatory T cells. Clinical cancer research : an official journal of the American Association for Cancer Research. 2008;14(8):2413-20.
[16] Ladoire S, Mignot G, Dabakuyo S, Arnould L, Apetoh L, Rebe C, et al. In situ immune response after neoadjuvant chemotherapy for breast cancer predicts survival. The Journal of pathology. 2011;224(3):389-400.
[17] Lee J, Kim DM, Lee A. Prognostic Role and Clinical Association of Tumor-Infiltrating Lymphocyte, Programmed Death Ligand-1 Expression with Neutrophil-Lymphocyte Ratio in Locally Advanced Triple-Negative Breast Cancer. Cancer research and treatment. 2019;51(2):649-63.
[18] Li X, Warren S, Pelekanou V, Wali V, Cesano A, Liu M, et al. Immune profiling of pre- and post-treatment breast cancer tissues from the SWOG S0800 neoadjuvant trial. Journal for immunotherapy of cancer. 2019;7(1):88.
[19] Liang H, Huang J, Ao X, Guo W, Chen Y, Lu D, et al. TMB and TCR Are Correlated Indicators Predictive of the Efficacy of Neoadjuvant Chemotherapy in Breast Cancer. Frontiers in oncology. 2021;11:740427.
[20] Miyashita M, Sasano H, Tamaki K, Hirakawa H, Takahashi Y, Nakagawa S, et al. Prognostic significance of tumor-infiltrating CD8+ and FOXP3+ lymphocytes in residual tumors and alterations in these parameters after neoadjuvant chemotherapy in triple-negative breast cancer: a retrospective multicenter study. Breast cancer research : BCR. 2015;17:124.
[21] Nadin SB, Sottile ML, Montt-Guevara MM, Gauna GV, Daguerre P, Leuzzi M, et al. Prognostic implication of HSPA (HSP70) in breast cancer patients treated with neoadjuvant anthracycline-based chemotherapy. Cell stress & chaperones. 2014;19(4):493-505.
[22] Oda N, Shimazu K, Naoi Y, Morimoto K, Shimomura A, Shimoda M, et al. Intratumoral regulatory T cells as an independent predictive factor for pathological complete response to neoadjuvant paclitaxel followed by 5-FU/epirubicin/cyclophosphamide in breast cancer patients. Breast cancer research and treatment. 2012;136(1):107-16.
[23] Pelekanou V, Barlow WE, Nahleh ZA, Wasserman B, Lo YC, von Wahlde MK, et al. Tumor-Infiltrating Lymphocytes and PD-L1 Expression in Pre- and Posttreatment Breast Cancers in the SWOG S0800 Phase II Neoadjuvant Chemotherapy Trial. Molecular cancer therapeutics. 2018;17(6):1324-31.
[24] Sarradin V, Lusque A, Filleron T, Dalenc F, Franchet C. Immune microenvironment changes induced by neoadjuvant chemotherapy in triple-negative breast cancers: the MIMOSA-1 study. Breast cancer research : BCR. 2021;23(1):61.
[25] Uruena C, Lasso P, Bernal-Estevez D, Rubio D, Salazar AJ, Olaya M, et al. The breast cancer immune microenvironment is modified by neoadjuvant chemotherapy. Scientific reports. 2022;12(1):7981.
[26] Vanguri RS, Fenn KM, Kearney MR, Wang Q, Guo H, Marks DK, et al. Tumor Immune Microenvironment and Response to Neoadjuvant Chemotherapy in Hormone Receptor/HER2+ Early Stage Breast Cancer. Clinical breast cancer. 2022;22(6):538-46.
[27] Varadan V, Gilmore H, Miskimen KL, Tuck D, Parsai S, Awadallah A, et al. Immune Signatures Following Single Dose Trastuzumab Predict Pathologic Response to PreoperativeTrastuzumab and Chemotherapy in HER2-Positive Early Breast Cancer. Clinical cancer research : an official journal of the American Association for Cancer Research. 2016;22(13):3249-59.

[28] Verma C, Kaewkangsadan V, Eremin JM, Cowley GP, Ilyas M, El-Sheemy MA, et al. Natural killer (NK) cell profiles in blood and tumour in women with large and locally advanced breast cancer (LLABC) and their contribution to a pathological complete response (PCR) in the tumour following neoadjuvant chemotherapy (NAC): differential restoration of blood profiles by NAC and surgery. Journal of translational medicine. 2015;13:180.
[29] Waks AG, Stover DG, Guerriero JL, Dillon D, Barry WT, Gjini E, et al. The Immune Microenvironment in Hormone Receptor-Positive Breast Cancer Before and After Preoperative Chemotherapy. Clinical cancer research : an official journal of the American Association for Cancer Research. 2019;25(15):4644-55.
[30] Wang Y, Dong T, Xuan Q, Zhao H, Qin L, Zhang Q. Lymphocyte-Activation Gene-3 Expression and Prognostic Value in Neoadjuvant-Treated Triple-Negative Breast Cancer. Journal of breast cancer. 2018;21(2):124-33.
[31] Wesolowski R, Stiff A, Quiroga D, McQuinn C, Li Z, Nitta H, et al. Exploratory analysis of immune checkpoint receptor expression by circulating T cells and tumor specimens in patients receiving neo-adjuvant chemotherapy for operable breast cancer. BMC cancer. 2020;20(1):445.
[32] Zhang L, Wang XI, Ding J, Sun Q, Zhang S. The predictive and prognostic value of Foxp3+/CD25+ regulatory T cells and PD-L1 expression in triple negative breast cancer. Annals of diagnostic pathology. 2019;40:143-51.
