# Supplementary material for: Effect of neoadjuvant chemotherapy on tumor immune infiltration in breast cancer patients: Systematic review and meta-analysis
Source: PLoS One. 2023 Apr 27;18(4):e0277714. doi: 10.1371/journal.pone.0277714 (PMC10138237; doi:10.1371/journal.pone.0277714)
Supplement: S4 Table — (PDF) [file pone.0277714.s008.pdf]

SUPPLEMENTARY MATERIAL 4

Table S4. TILs trends	
**Article**	**TILs tendency**
Abdel-Fatah 2014 [1]	= NR-p
Abdelrahman, 2021 [2]	= NS-p
Alhesa 2022 [3]	= NS-p
Demaria 2001 [4]	= NS-p
Dieci 2020 [5]	= NS-p
Grecco-Hoffman 2021 [6]	= NS-p
Hee Park 2020 [7]	⇩**
Kaewkangsadan 2016 [8]	Responders = NS-p<br>Non-responders = NS-p
Lee 2019 [9]	⇧ NR-p
Li 2019 [10]	Responders: ⇩***<br>Non-Responders = NS-p
Nadin 2014 [11]	Stromal ⇧**<br>Tumoral ⇧***
Pelekanou 2018 [12]	⇩ NR- p
Sarradin 2021 [13]	= NS-p
Urueña, 2022 [14]	Stromal = NS-p<br>Tumoral = NS-p
Verma 2015 [15]	= NR-p
Waks 2019 [16]	= NS-p
Wesolowski 2020 [17]	= NR-p
* p0.05, **p0.01, ***p0.001; NS-p: P value not significant; NR-p: P value not reported; NM: Not measured	

1. Abdel-Fatah, T.M., et al., *HAGE (DDX43) is a biomarker for poor prognosis and a predictor of chemotherapy response in breast cancer.* Br J Cancer, 2014. **110**(10): p. 2450-61.
2. Abdelrahman, A.E., et al., *Clinicopathological significance of the immunologic signature (PDL1, FOXP3+ Tregs, TILs) in early stage triple-negative breast cancer treated with neoadjuvant chemotherapy.* Ann Diagn Pathol, 2021. **51**: p. 151676.
3. Alhesa, A., et al., *PD-L1 expression in breast invasive ductal carcinoma with incomplete pathological response to neoadjuvant chemotherapy.* International journal of immunopathology and pharmacology, 2022. **36**.

4. S, Demaria, et al., *Development of tumor-infiltrating lymphocytes in breast cancer after neoadjuvant paclitaxel chemotherapy.* Clinical cancer research : an official journal of the American Association for Cancer Research, 2001. **7**(10).
5. Dieci, M., et al., *Integration of tumour infiltrating lymphocytes, programmed cell-death ligand-1, CD8 and FOXP3 in prognostic models for triple-negative breast cancer: Analysis of 244 stage I-III patients treated with standard therapy.* European journal of cancer (Oxford, England : 1990), 2020. **136**.
6. Hoffmann, L.G., et al., *Evaluation of PD-L1 and tumor infiltrating lymphocytes in paired pretreatment biopsies and post neoadjuvant chemotherapy surgical specimens of breast carcinoma.* Sci Rep, 2021. **11**(1): p. 22478.
7. YH, Park., et al., *Chemotherapy induces dynamic immune responses in breast cancers that impact treatment outcome.* Nature communications, 2020. **11**(1).
8. V, Kaewkangsadan., et al., *Crucial Contributions by T Lymphocytes (Effector, Regulatory, and Checkpoint Inhibitor) and Cytokines (TH1, TH2, and TH17) to a Pathological Complete Response Induced by Neoadjuvant Chemotherapy in Women with Breast Cancer.* Journal of immunology research, 2016. **2016**.
9. Lee, J., D. Kim, and A. Lee, *Prognostic Role and Clinical Association of Tumor-Infiltrating Lymphocyte, Programmed Death Ligand-1 Expression with Neutrophil-Lymphocyte Ratio in Locally Advanced Triple-Negative Breast Cancer.* Cancer research and treatment, 2019. **51**(2).
10. Li, X., et al., *Immune profiling of pre- and post-treatment breast cancer tissues from the SWOG S0800 neoadjuvant trial.* Journal for immunotherapy of cancer, 2019. **7**(1).
11. Nadin, S., et al., *Prognostic implication of HSPA (HSP70) in breast cancer patients treated with neoadjuvant anthracycline-based chemotherapy.* Cell stress & chaperones, 2014. **19**(4).
12. Pelekanou, V., et al., *Tumor-Infiltrating Lymphocytes and PD-L1 Expression in Pre- and Posttreatment Breast Cancers in the SWOG S0800 Phase II Neoadjuvant Chemotherapy Trial.* Molecular cancer therapeutics, 2018. **17**(6).
13. Sarradin, V., et al., *Immune microenvironment changes induced by neoadjuvant chemotherapy in triple-negative breast cancers: the MIMOSA-1 study.* Breast cancer research : BCR, 2021. **23**(1).
14. Urueña, C., et al., *The breast cancer immune microenvironment is modified by neoadjuvant chemotherapy.* Scientific reports, 2022. **12**(1).
15. Verma, C., et al., *Natural killer (NK) cell profiles in blood and tumour in women with large and locally advanced breast cancer (LLABC) and their contribution to a pathological complete response (PCR) in the tumour following neoadjuvant chemotherapy (NAC): differential restoration of blood profiles by NAC and surgery.* Journal of translational medicine, 2015. **13**.
16. Waks, A., et al., *The Immune Microenvironment in Hormone Receptor-Positive Breast Cancer Before and After Preoperative Chemotherapy.* Clinical cancer research : an official journal of the American Association for Cancer Research, 2019. **25**(15).
17. Wesolowski, R., et al., *Exploratory analysis of immune checkpoint receptor expression by circulating T cells and tumor specimens in patients receiving neo-adjuvant chemotherapy for operable breast cancer.* BMC Cancer, 2020. **20**(1): p. 445.
