# Supplementary material for: Effect of neoadjuvant chemotherapy on tumor immune infiltration in breast cancer patients: Systematic review and meta-analysis
Source: PLoS One. 2023 Apr 27;18(4):e0277714. doi: 10.1371/journal.pone.0277714 (PMC10138237; doi:10.1371/journal.pone.0277714)
Supplement: S5 Table — (PDF) [file pone.0277714.s009.pdf]

SUPPLEMENTARY MATERIAL 5

**Table S5. Cells trends**	
**Article**	**Cell tendency**
Abdelrahman, 2021 (33360026)	FoxP3 ⇩**
Chan 2014 (24803281)	CD8 **Responders** ⇧***<br>CD8 **Non-Responders**⇧***<br>FoxP3 **Responders** ⇩***<br>FoxP3 **Non-Responders** ⇩ **NS-p**<br>CD8/FoxP3 **Responders**⇧***<br>CD8/FoxP3 **Non-Responders**⇧***
Demaria 2001 (11595690)	CD3 **=NR-p**
Demir 2013 (11595690)	FoxP3 ⇩***
Dieci 2020 (32622323)	CD8 ⇧*<br>FoxP3 **=NS-p**
García-Martínez 2014 (25432519)	CD3 **=NS-p**<br>CD4 ⇩**<br>CD8 ⇧***<br>FoxP3 **=NS-p**<br>LB ⇩*<br>M2 ⇩*
Graeser 2021 (25432519)	Stroma<br>CD4+ ⇧***<br>CD8+ ⇧***<br>T cells ⇧***<br>CD4+/CD8+ **= NS-p**<br><br>Tumor<br>CD4+ ⇧***<br>CD8+ ⇧***<br>T cells ⇧***<br>CD4+/CD8+ **= NS-p**
Hornychova 2008 (19093260)	Stromal CD3 ⇧*<br>Intraepithelial CD3 ⇧**<br>Stromal NK ⇧**<br>Intraepithelial NK **=NS-p**<br>Stromal M2 ⇩**<br>Intraepithelial M2 **=NS-p**<br>Stromal DCs(CD1a) ⇧*<br>Intraepithelial DCs (CD1a)**=NS-p**<br>Stromal DCs(S100) ⇧*<br>Intraepithelial DCs (S100) **=NS-p**<br>StromalDCs(CD83)⇧***<br>Intraepithelial DCs (CD83) **=NS-p**
Kaewkangsadan 2016 (33268821)	Stromal CD4 ⇧**<br>Tumoral CD4 ⇧**<br>Stromal CD8 **=NS-p**<br>Tumoral CD8 **=NS-p**

	Stromal FoxP3 ⇩***<br>Tumoral FoxP3 ⇩***
Kaewkangsadan 2017 (28913366)	Stromal TINs =NS-p<br>Intratumoral TINs =NS-p<br>M1 =NR-p<br>M2 =NR-p<br>Stromal DCs (CD1a) =NS-p<br>Intratumoral DCs (CD1a) ⇧*
Ladoire 2008 (28913366)	**CD3 =NR-p**<br>**CD8 =NR-p**<br>**FoxP3 ⇩ NR-p**
Ladoire 2011 (21437909)	CD8 =NS-p<br>FoxP3 ⇩ NR-p<br>CD8/FoxP3 =NR-p
Lee 2019 (30064200)	CD4 ⇧ NR-p<br>CD8 ⇧ NR-p<br>FoxP3 ⇩ NR-p<br>CD8/FoxP3 ⇧ NR-p
Liang 2021 (34950580)	TMB ⇩**<br>DC50 =NS-p<br>CD3 ⇧*<br>CD4 =NS-p<br>CD8 ⇧*<br>CD4/CD8 ⇩*
Miyashita 2015 (26341640)	CD8 ⇧ NR-p<br>FoxP3 =NR-p<br>CD8/FoxP3 =NR-p
Naofumi Oda 2012 (22986814)	CD8 ⇩***<br>FoxP3 ⇩***
Urueña, 2022 (35562400)	CD45 =NS-p<br>CD3 =NS-p<br>CD4 =NS-p<br>CD8 =NS-p<br>CD20 =NS-p<br>FoxP3 =NS-p<br>CD68 =NS-p
Vanguri 2022 (35562400)	CD3 =NS-p<br>CD3+CD8-FOXP3- =NR-p<br>CD68 =NS-p<br>CD8 ⇩***<br>FoxP3 ⇩*
Varadan 2016 (26842237)	Immune index =NS-p
Verma 2015 (26040463)	NK =NR-p
Waks 2019 (31061067)	CD8 = NR-p<br>FoxP3 ⇩ NR-p<br>M1 ⇧*<br>M2 ⇧*/⇩*<br>DCs ⇧***
Wang 2018 (29963107)	**CD8⇧******

Wesolowski 2020 (32429929)	Overall CD8 **= NR-p**<br>Intratumoral CD8 **= NR-p**
Zhang 2019 (31096176)	FoxP3 ⇩**
* p0.05, **p0.01, ***p0.001; NS-p: P value not significant; NR-p: P value not reported; NM: Not measured	
