# Supplementary material for: Effect of neoadjuvant chemotherapy on tumor immune infiltration in breast cancer patients: Systematic review and meta-analysis
Source: PLoS One. 2023 Apr 27;18(4):e0277714. doi: 10.1371/journal.pone.0277714 (PMC10138237; doi:10.1371/journal.pone.0277714)
Supplement: S6 Table — (PDF) [file pone.0277714.s010.pdf]

SUPPLEMENTARY MATERIAL 6

Table S6. Cytokines and markers trends	
**Article**	**Cytokine or marker tendency**
Abdelrahman, 2021 (33360026)	**PD-L1** = NS-p
Graeser 2021 (25432519)	Stroma<br>PD1 en CD4 = NS-p<br>PD1 en CD8 ⇧**<br>PD-L1 = NS-p<br><br>Tumor<br>PD1 en CD4 = NS-p<br>PD1 en CD8 ⇧***<br>PD-L1 ⇧**
Grecco-Hoffman 2021 (34795307)	**PD-L1** = NS-p
Kaewkangsadan 2016 (33268821)	PD-L1 = NS-p<br>Stromal CTLA-4 ⇩*<br>Tumoral CTLA-4 = NS-p<br>Stromal PD-1 ⇩**<br>Tumoral PD-1 ⇩**<br>IL-1 = NS-p<br>IL-2 = NS-p<br>IL-4 ⇩**<br>IL-10 = NS-p<br>IL-17 = NS-p<br>IFN-y = NS-p<br>TGF-B = NS-p
Lee 2019 (30064200)	Stromal PD-L1 ⇧***<br>Tumoral PD-L1 =NS-p
Liang 2021 (34950580)	PD-1 =NS-p<br>TIM3 =NS-p<br>CD8 PD1 ⇧*<br>CD4 PD1 =NS-p<br>CD8 TIM3 =NS-p<br>CD4 TIM3 =NS-p
Naofumi Oda 2012 (22986814)	**IL-17F** =NR-p
Pelekanou 2018 (29588392)	PD-L1 = NS-p
Sarradin 2021 (34039396)	PD-L1 =NS-p<br>TIM3 ⇧***<br>LAG3 ⇩*
Verma 2015 (26040463)	TGFb = NR-p<br>IFNg = NR-p<br>IL-2 = NR-p
Wang 2018 (29963107)	PD-L1 = NS-p<br>PD-1 ⇧*<br>LAG-3 ⇧*
Wesolowski 2020 (32429929)	Overall PD-L1 = NR-p<br>Stromal PD-L1 = NR-p

	Intratumoral PD-L1 **= NR-p**<br>Overall PD-1 **= NR-p**
Zhang 2019 (31096176)	PD-L1 Combined Positive Score **⇩****<br>Tumoral cells PD-L1 **= NR-p**<br>PD-L1 TILS **= NR-p**
* p0.05, **p0.01, ***p0.001; NS-p: P value not significant; NR-p: P value not reported; NM: Not measured	
