# Supplementary material for: Effect of neoadjuvant chemotherapy on tumor immune infiltration in breast cancer patients: Systematic review and meta-analysis
Source: PLoS One. 2023 Apr 27;18(4):e0277714. doi: 10.1371/journal.pone.0277714 (PMC10138237; doi:10.1371/journal.pone.0277714)
Supplement: Attachment — Submitted filename: Plos one-reviewers comments.docx [file pone.0277714.s011.docx]

**PONE-D-22-27675**

**"Effect of neoadjuvant chemotherapy on tumor immune infiltration in breast cancer patients: systematic review and meta-analysis."**

Tumor microenvironments are complex structures comprised of various interconnected layers that are difficult to define and study. The tumor immune microenvironment (TIME), in particular, refers to the presence of leukocytes, as well as their products, surface markers, and gene expression profiles within or around a tumor. The variability in the presence, location, and functional organization of infiltrating immune cells suggests that different populations may play distinct roles in the control or promotion of tumor growth. The study of these characteristics has led to the identification of prognostic markers for clinical response to treatment, resulting in the development of different approaches to classify neoplasms. In recent years, the validation of models such as Immunoscore has allowed for a more refined evaluation of patients' prognoses, particularly in colorectal cancer. In breast cancer (BC), the importance of immune infiltration as a prognostic factor for clinical response to neoadjuvant chemotherapy (NAC) has also been evaluated. Chemotherapy has traditionally been seen as an immunosuppressive treatment, but evidence suggests that it can actually activate the immune system by inducing immunogenic cell death in tumor cells, leading to the release of DAMPs.

This review aims to determine whether there are changes in the cellular and molecular components of the immune infiltrate in BC tumours in response to NAC. A systematic search of the literature was performed, and studies comparing immune molecules or cells before and after treatment were included. The results showed that NAC leads to an increase in the expression of immune molecules such as HLA-DR, PD-L1, and CD8, as well as a decrease in the expression of immune inhibitory molecules such as PD-1, CTLA-4, and Tim-3. There was also an increase in the infiltration of immune cells such as T lymphocytes, dendritic cells, and macrophages in response to NAC. However, these findings are not universal and are not present in all studies. Some papers showed there is no correlation between immune cells and NAC, while others showed the opposite result. Due to the heterogeneity among the studies, there is no conclusive evidence that suggests NAC can lead to a more favourable.

Liano-Leon et al addressed an important question, though there is no conclusion. It is still important to consider this topic for future study designs. However, although they thoroughly checked most of the available literature, I would not recommend accepting the current form of the manuscript. If the work is to be accepted for publication, I suggest addressing the following points:

**Major Comments:**

1. The English of the manuscript is generally acceptable, but the structure of the manuscript could be improved to make it more reader-friendly, particularly in the Results and Discussion sections. Those can be written in a more concise way. There is a lot of redundancy in the Discussion and Results sections. The authors could consider cutting and pasting some text from the Discussion section to the Results section in order to improve the overall structure and clarity of the paper. It may be helpful to include some background information on the cells or molecules being studied in the Results section, as this can provide context and make the results more meaningful for readers who are not familiar with them. Additionally, it would be beneficial to include an overall conclusion or implication of the results at the end of the Results section. This could highlight any trends observed or speculations that can be made based on the data.
2. Line 150-157: The Table 1 can be found in the supplementary section. A Venn diagram can provide a visual representation of the table's content, which may be more appealing to readers than a lengthy table.
3. Line 191- 216: Table 2, which describs all the TILs’ results can be moved to the supplementary section. Forest plots or funnel plots may be easy more easily understood by the readers.
4. Similarly, Table 3 can be moved to the supplementary section and forest plots or funnel plots may be more easily understood by readers.
5. The authors concluded that there was a significant decrease in TILs (tumor infiltrating lymphocytes) and Foxp3 expression after NAC. I am wondering if TILs and Foxp3 can be used as prognostic markers for DFS after NAC. Is it possible to develop a logistic regression equation using these variables?

**Minor Comments:**

1. Fig 2 is enough for main figure. Fig 3 can be found in the supplementary section. The description of a-m should be in Fig 2 legend.
2. Line 233: What is NOtcWEh1? Is it a typo of Notch1? I did not find it in the reference (49).
3. Line 110-IFN-y, Line 50: IFNg. Are authors mentioning IFN-gamma (IFNg) at Line 110?
