# Supplementary material for: Effect of neoadjuvant chemotherapy on tumor immune infiltration in breast cancer patients: Systematic review and meta-analysis
Source: PLoS One. 2023 Apr 27;18(4):e0277714. doi: 10.1371/journal.pone.0277714 (PMC10138237; doi:10.1371/journal.pone.0277714)
Supplement: Attachment — Submitted filename: Response to reviewers.docx [file pone.0277714.s012.docx]

Bogota, February 28, 2023

**Review:**

**PONE-D-22-27675**

**"Effect of neoadjuvant chemotherapy on tumor immune infiltration in breast cancer patients: systematic review and meta-analysis."**

**PLOS ONE**

Dear academic editor and reviewer(s),

In accordance with your observations and suggestions, we allow ourselves to respond to the following points through the next table:

**Reviewer 1 comments**:

1. *Please provide a detailed description of the “Search strategy” so that it is understandable to a broader audience.*

We have described in more detail the Search strategy in lines 81-87 in the non-tracked version.

2. *In Figure 2 legend, a), b), c), ….m) are not specified. I assume those are same as that of Figure3, please clarify.*

We describe the *a* to *m* variables in the figure 2.

3. *Figure 3 shows the same results as that of Figure 2, only those are represented article-wise, without any substantial new information. Therefore, this figure 3 can go as a Supplementary figure.*

We change Figure 3 to Figure S1.

4. *In table-2, instead of presenting which article shows what TIL trend, it would be easier to understand if the authors present which TIL trend (increase, decrease, no change) appears in how many studies with a simple bar graph.*

Table 2 were replaced with table 1. This table includes bar graphs presenting TILs and cells trends.

5. *Too many information are spread across Table-3 which makes it difficult to comprehend. Again, instead of showing cell type trends in each article separately, it would be easier to follow if the authors show which cell type trend appears in how many studies with a graph if possible.*

Table 3 were replaced with table 1. This table includes bar graphs presenting TILs and cells trends.

6. *Since the present study has been limited only to neoadjuvant chemotherapy, very few studies qualified for meta-analysis. It might provide a broader picture if other neoadjuvant therapies (immunotherapy, hormone therapy etc.) are included and the findings may be clinically more significant.*

The aim of this review was to evaluate the effect of neoadjuvant chemotherapy on the tumor immune infiltrate; therefore we defined the combination with other therapies as an exclusion criteria. Including these therapies would imply carrying out another search strategy that would not meet our aim. However, we will consider it for future works, as the reviewer's comment seems very relevant to us.

**Reviewer 2 comments**:

**Major comments:**

1. *The English of the manuscript is generally acceptable, but the structure of the manuscript could be improved to make it more reader-friendly, particularly in the Results and Discussion sections. Those can be written in a more concise way. There is a lot of redundancy in the Discussion and Results sections. The authors could consider cutting and pasting some text from the Discussion section to the Results section in order to improve the overall structure and clarity of the paper. It may be helpful to include some background information on the cells or molecules being studied in the Results section, as this can provide context and make the results more meaningful for readers who are not familiar with them. Additionally, it would be beneficial to include an overall conclusion or implication of the results at the end of the Results section. This could highlight any trends observed or speculations that can be made based on the data.*

We move sections of the discussion to the results and made a brief description of the cells and markers evaluated. The new texts are found in the following lines:

- TILs: lines 195-196
- Macrophages: lines 244-246
- Neutrophils: lines 253-255
- Natural killer cells: lines 261-263
- Dendritic cells: lines 292-295
- B cells: lines 287-290
- T cells: lines 297-302
- CD4 T cells: lines 321-323
- CD8 T cells: lines 342-344
- FoxP3: lines 383-385
- Immune checkpoint and cytokines: lines 417-427; lines 460-472; lines 476-478.

Finally, we added a conclusion paragraph in lines 499-510 at the end of the result section.

2. *Line 150-157: The Table 1 can be found in the supplementary section. A Venn diagram can provide a visual representation of the table's content, which may be more appealing to readers than a lengthy table.*

We replace Table 1 with a waffle plot where each box represents an article which measure a type of cell or a marker. This table was move to the supplementary information as Table S1.

3. *Line 191- 216: Table 2, which describs all the TILs’ results can be moved to the supplementary section. Forest plots or funnel plots may be easy more easily understood by the readers.*

Table 2 were replaced with table 1. This table includes bar graphs presenting TILs and cells trends.

4. *Similarly, Table 3 can be moved to the supplementary section and forest plots or funnel plots may be more easily understood by readers.*

Table 3 were replaced with table 1. This table includes bar graphs presenting TILs and cells trends.

5. *The authors concluded that there was a significant decrease in TILs (tumor infiltrating lymphocytes) and Foxp3 expression after NAC. I am wondering if TILs and Foxp3 can be used as prognostic markers for DFS after NAC. Is it possible to develop a logistic regression equation using these variables?*

Quantitative data was extracted directly from the graphs through the Graph grabber extraction program, since this information was requested to the authors of each article without receiving any response. Therefore, we do not have the raw data paired with the clinical outcome of the patients and we cannot perform a meta-regression to define the prognostic value of these variables.

**Minor comments:**

1. *Fig 2 is enough for main figure. Fig 3 can be found in the supplementary section. The description of a-m should be in Fig 2 legend.*

We describe the *a* to *m* variables in the figure 2 and move Figure 3 to the supplementary information as Figure S1.

2. *Line 233: What is NOtcWEh1? Is it a typo of Notch1? I did not find it in the reference (49).*

NotcWEh1 was a typo, we corrected it to Notch1.

3. *Line 110-IFN-y, Line 50: IFNg. Are authors mentioning IFN-gamma (IFNg) at Line.*

We change all forms of IFN-gamma to IFNg.

Thank you to the reviewers for their valuable feedback. We have carefully considered and incorporated your comments and look forward to hearing back from the editors regarding the final decision on our manuscript.

Sincerely,

*Carlos Alberto Parra López MD., Ph. D.*

*Full Professor School of Medicine*

*Microbiology Department*

*Universidad Nacional de Colombia*
